# Electrical Responses and Spontaneous Activity of Human iPS-Derived Neuronal Networks Characterized for 3-month Culture with 4096-Electrode Arrays

**DOI:** 10.3389/fnins.2016.00121

**Published:** 2016-03-30

**Authors:** Hayder Amin, Alessandro Maccione, Federica Marinaro, Stefano Zordan, Thierry Nieus, Luca Berdondini

**Affiliations:** ^1^NetS^3^ Laboratory, Neuroscience and Brain Technologies Department, Fondazione Istituto Italiano di Tecnologia (IIT)Genova, Italy; ^2^Neurobiology of miRNA Laboratory, Neuroscience and Brain Technologies Department, Fondazione Istituto Italiano di Tecnologia (IIT)Genova, Italy

**Keywords:** iPSC-derived neurons, spontaneous and evoked activities, CMOS-multielectrode arrays, neural networks, surface functionalization

## Abstract

The recent availability of human induced pluripotent stem cells (hiPSCs) holds great promise as a novel source of human-derived neurons for cell and tissue therapies as well as for *in vitro* drug screenings that might replace the use of animal models. However, there is still a considerable lack of knowledge on the functional properties of hiPSC-derived neuronal networks, thus limiting their application. Here, upon optimization of cell culture protocols, we demonstrate that both spontaneous and evoked electrical spiking activities of these networks can be characterized on-chip by taking advantage of the resolution provided by CMOS multielectrode arrays (CMOS-MEAs). These devices feature a large and closely-spaced array of 4096 simultaneously recording electrodes and multi-site on-chip electrical stimulation. Our results show that networks of human-derived neurons can respond to electrical stimulation with a physiological repertoire of spike waveforms after 3 months of cell culture, a period of time during which the network undergoes the expression of developing patterns of spontaneous spiking activity. To achieve this, we have investigated the impact on the network formation and on the emerging network-wide functional properties induced by different biochemical substrates, i.e., poly-dl-ornithine (PDLO), poly-l-ornithine (PLO), and polyethylenimine (PEI), that were used as adhesion promoters for the cell culture. Interestingly, we found that neuronal networks grown on PDLO coated substrates show significantly higher spontaneous firing activity, reliable responses to low-frequency electrical stimuli, and an appropriate level of PSD-95 that may denote a physiological neuronal maturation profile and synapse stabilization. However, our results also suggest that even 3-month culture might not be sufficient for human-derived neuronal network maturation. Taken together, our results highlight the tight relationship existing between substrate coatings and emerging network properties, i.e., spontaneous activity, responsiveness, synapse formation and maturation. Additionally, our results provide a baseline on the functional properties expressed over 3 months of network development for a commercially available line of hiPSC-derived neurons. This is a first step toward the development of functional pre-clinical assays to test pharmaceutical compounds on human-derived neuronal networks with CMOS-MEAs.

## Introduction

Brain diseases represent a major challenge in neuroscience. Many of these neurodegenerative disorders are still missing disease-specific therapies, while the long life expectancy of our society is constantly increasing the impact of these daunting diseases.

Advances in drug-discovery and therapeutic research and development (R&D) are still facing a limited understanding of normal brain function and disease mechanisms. In bio/-pharmaceutical R&D, the pre-clinical phase of drug discovery, is a recognized obstacle, resulting in a high economic burden and in slowing down therapeutic R&D (Orloff et al., 2009). In particular, toxicity and efficacy of molecular drug candidates are often issues, lately identified in clinical trials. Among the different reasons of this situation, the failure in translating pre-clinical results from animal testing to human is considered part of the problem.

An emerging possible way to accelerate research on brain diseases relies on the development of assays that exploit the recent availability of human-derived neurons. These neurons arise from the development of induced pluripotent stem cells (iPSCs) derived from human cells (Takahashi et al., 2007). Following this early achievement, the iPS technology is nowadays attracting a growing interest for studying the pathogenesis of a wide range of human diseases, for investigating the use of derived human cells to grow *in vitro* models for pharmacological and toxicological testing as well as for research on potential cell-based therapeutic approaches (Espuny-Camacho et al., 2013).

Importantly, in order to be able to exploit the iPS technology for all these potential applications with electrogenic human-derived cells, an important prerequisite is the assessment of their cellular and network-wide electrophysiological properties. Over the last years different laboratories have been examining the electrophysiological responses of different hiPSCs to pharmaceutical compounds. In particular, studies on hiPSC-derived cardiomyocytes have been remarkably advanced. Hence, as reviewed in Rajamohan et al. (2013) nearly 60 compounds have been quantified for drug screening by using patch-clamping and conventional multielectrode arrays (MEAs) to interrogate the electrophysiological properties and responses of these cell cultures. Only recently, stable lines of hiPSC–derived neurons have become commercially available. Therefore, large pure quantities of differentiated human neurons are now accessible and offer a unique opportunity for developing standardized *in vitro* assays for drugs targeting central nervous system (CNS) disorders.

The cell culture conditions and the spontaneous activity emerging during *in vitro* network development of a commercially available line of hiPSC–derived neurons, i.e., iCell neurons, was recently investigated on-chip by using conventional MEAs recording from 60 electrodes (Odawara et al., 2014). These devices represent a well-established technology, as demonstrated for recording network-wide extracellular activity from neuronal cultures prepared from rodents; for studying the developing patterns of network activity (Chiappalone et al., 2006; Wagenaar et al., 2006) and to investigate the network responsiveness to electrical stimuli (Jimbo and Kawana, 1992; Wagenaar et al., 2004; Ide et al., 2010). Interestingly, the iCell neurons used in the study of Odawara and co-authors are a valuable cell line that counts on a wealth of data describing their cellular, molecular, and functional properties as acquired with different methodologies (Haythornthwaite et al., 2012; Whitemarsh et al., 2012; Gill et al., 2013; Dage et al., 2014; Chatzidaki et al., 2015). However, the capability of these neuronal networks to respond to electrical stimuli was not yet assessed and the developmental patterns of spiking activity still lack of detailed characterization reports. Additionally, long-term culture conditions still need to be optimized.

In the present study, we investigate both spontaneous activity and responsiveness to electrical stimuli of iCell neuronal networks grown under different cell culture conditions. In particular, we examine whether iCell neuronal networks can develop over 3 months without the use of glial co-cultures or conditioned media, as previously reported (Odawara et al., 2014), by optimizing the biochemical substrate and by studying the induced effects on the network development. Indeed, it is known that in order to promote cellular adhesion, growth, and formation of functional networks of primary neuronal cultures, appropriate biochemical substrates that consist in polycationic adhesion promoting molecules are needed. Here, we investigate three different coating reagents, i.e., poly-dl-ornithine (PDLO), poly-l-ornithine (PLO), and polyethylenimine (PEI), all supported with laminin, a component of the extracellular matrix that was previously reported to promote neurite axonal growth (Matsuzawa et al., 1996).

To perform multi-site extracellular recordings and to deliver electrical stimuli, we exploit an emerging generation of high-resolution MEAs that can simultaneously records extracellular spiking activity from 4096 electrodes (Berdondini et al., 2001, 2009) and that provide distinct sites for electrical stimulation. This device is realized with complementary-metal-oxide-semiconductor (CMOS) technology and, differently than other similar developments (Eversmann et al., 2003; Franks et al., 2003), the CMOS-MEAs used in this work integrate on-chip circuits for sub-millisecond recordings from the entire array. As reported in our previous work on primary neuronal cultures from rodents (Maccione et al., 2010), this recoding capability allows reduction of the under-sampling of neuronal activity in networks, i.e., 4096 vs. 60 simultaneously recording electrodes, and to improve the statistical significance of mean network-wide activity parameters, such as the mean firing rate for each recorded culture. Additionally, the high electrode density of these devices enables detecting only a few spiking neurons within the entire network. Therefore, this CMOS-MEA device can provide a detailed characterization of both spontaneous and evoked electrophysiological activities in networks of hiPSC–derived neurons.

**Figure 1:**
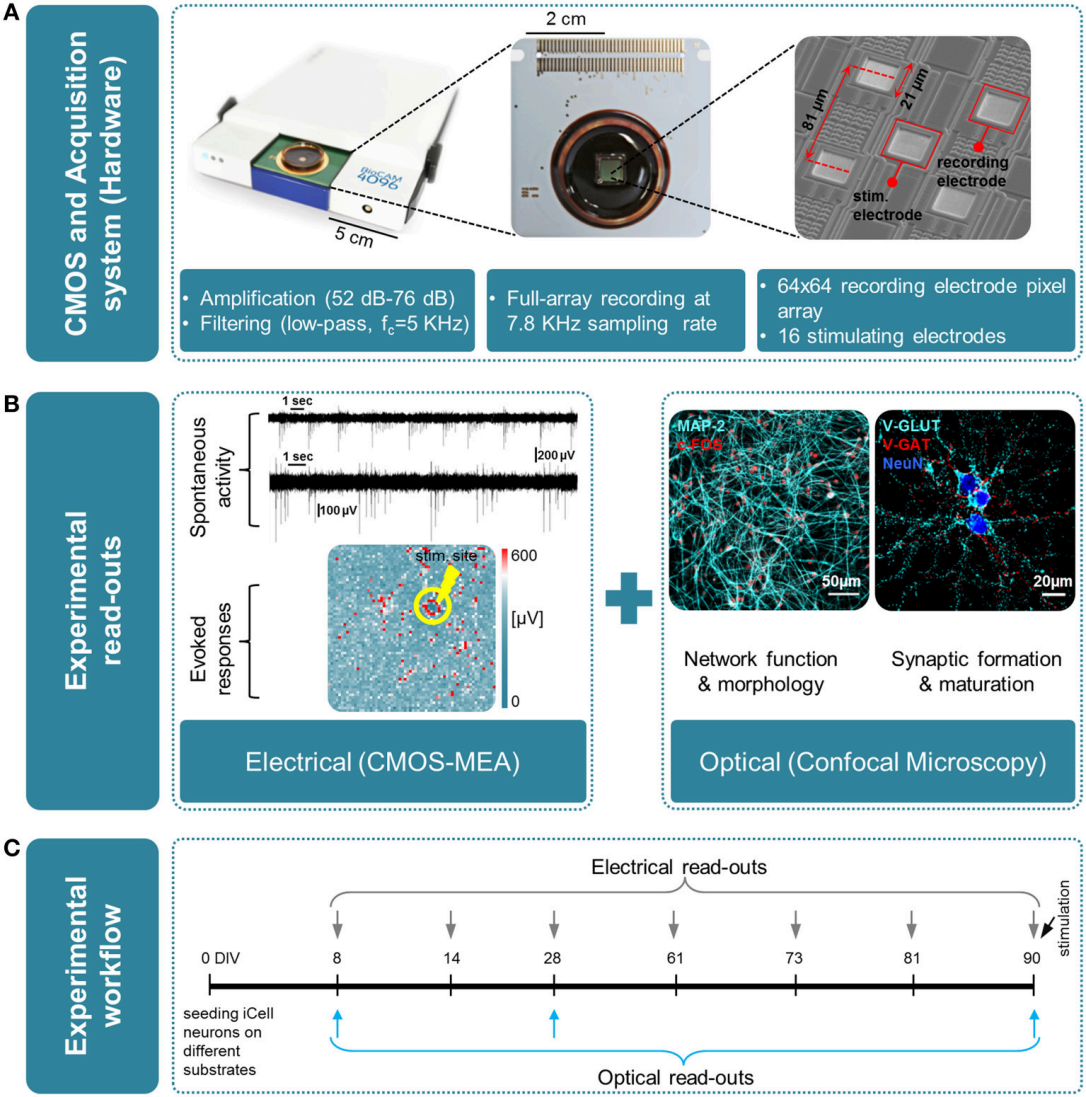
**Overview of the experimental platform used in this work for high-resolution electrical and optical read-outs**. (A)(from left to right); Real-time hardware for electrophysiological recordings from CMOS multielectrode arrays (CMOS-MEAs); view of a CMOS-MEA that features on-chip recording and stimulation electrodes (3Brain GmbH, Switzerland); scanning electron microscope (SEM) image of an area of the electrode array, with recording and stimulating electrodes. **(B)** Overview of the experimental read-outs used in this work, combining optical and electrical measures, i.e., left: electrical using CMOS-MEA; right: optical by means of confocal microscopy. **(C)** Time line of the experiment and time-points for optical and electrical measures.

As illustrated in Figure 1, electrical measures obtained from CMOS-MEAs are here completed with optical read-outs using confocal microscopy to document the growth, function, and synaptic maturation of these networks under different cell culture conditions up to 90 DIV. The maturation and formation of synapse connections are investigated by quantifying the expression level of PSD-95 protein and by correlating this measure with changes in the network activity, as obtained from CMOS-MEA recordings. Post-synaptic density-95 (PSD-95) is known as the major post-synaptic scaffold protein that is exclusively localized to glutamatergic synapses (Hunt et al., 1996), and was reported to drive synaptic maturation (Okabe et al., 1999; El-Husseini et al., 2000), and to promote synapse stability (Ehrlich et al., 2007). Studies carried out *in vitro* on murine neuronal cultures have also previously shown that levels of PSD-95 increase during development (Sans et al., 2000; Sun and Turrigiano, 2011), but such quantification was not yet reported for the development of human-derived neuronal networks.

## Materials and methods

### Preparation of hiPSC-derived neuronal cultures on CMOS-MEAs

Frozen stocks of hiPSC-derived neurons were obtained from (Cellular Dynamics International (CDI), USA) in vials containing at least 2.5 million plateable cells. Cells were thawed following the manufacturer's instructions and seeded at a density of 200,000 cells per CMOS-MEA chip (BioChip 4096S+ and 4096E, 3Brain GmbH, Switzerland). The BioChip 4096S+ integrates an array of 4096 recording electrodes (21 × 21 μm^2^ in size, 42 μm pitch) on an active area of (2.67 × 2.67 mm^2^) centered in a working area of (6 × 6 mm^2^). While the BioChip 4096E, integrates two interleaved arrays of 4096 recording electrodes (21 × 21 μm^2^ in size, 81 μm pitch) and 16 stimulation electrodes (21 × 21 μm^2^ in size, 1246 μm pitch) on an active area of (5.12 × 5.12 mm^2^) centered in (6 × 6 mm^2^). For the cellular growth, three different adhesion factors were tested for coating the CMOS-MEA surface, namely PDLO 50 μg/ml (Sigma P0671), PLO 0.01% (Sigma P4957), and PEI 0.1% (Sigma P3143) all dissolved in borate buffer (100 mM boric acid, 25 mM sodium tetraborate at PH 8.4) (all from Sigma-Aldrich, Italy). In brief, CMOS-MEAs were sterilized in 70% ethanol for 20 min, followed by bountifully four times washing with Double Distilled Water (DDW), then chips were preconditioned, i.e., incubated for 48 h with Complete Maintenance Medium. Afterwards, medium was aspirated and chips were immediately coated and incubated for 6 h at 37°C with 5% CO_2_, 95% O_2_ and saturated humidity. The adhesion factor was aspirated after incubation and each chip was rinsed 4 times with DDW and left overnight to dry under a sterile laminar flow. A vial containing 2.5 million plateable iCell neurons was placed in a 37°C water bath for exactly 3 min. Neurons were then transferred into a 50 ml centrifuge tube, and 1 ml of Complete Maintenance Medium was added slowly (drop-wise) to the cells. The Complete Maintenance Medium containing iCell maintenance and supplement media (from CDI, USA) in addition to 1% penicillin-streptomycin (Life Technologies, Italy). Cells were swirled gently and a further 8 ml of complete maintenance medium was added slowly to the centrifuge tube. Finally, Cells were re-suspended at 5000 cell/μl and 40 μl drop was seeded on each CMOS-MEA, this included 10 μg/ml of laminin (Sigma-Aldrich, Italy) that was added to the 8 ml cell solution. The chips were incubated at 37°C with 5% CO_2_ and 95% O_2_ and after 24 h, 100% cell culture medium was exchanged with Neurobasal Complete Medium that is comprised of (1x Neurobasal-A (NBA), 10% KnockOut Serum Replacement (KSR), and 1% penicillin-streptomycin (all from Life Technologies, Italy). Seeded CMOS-MEAs were afterward incubated at 37°C with 5% CO_2_ and 95% O_2_ for up to 12 weeks where a 50% medium exchange with Neurobasal Complete Medium was made every 4 days.

### Long-term, large-scale electrode array recordings, and analysis of spontaneous activity

Extracellular electrophysiological activity of *in vitro* spontaneously active hiPSC-derived neuronal networks grown on CMOS-MEAs was monitored over several months by using a custom platform build with components of the BioCam system (3Brain GmbH, Switzerland). Multiple recording sessions of 10 min were performed at full-frame resolution (at 7.8 KHz/electrode from 4096 electrodes), in addition to recordings from a selected region of interest (ROI) (at 22 KHz/electrode from 1024 electrodes). Experimental data (acquisition rate of 60 Mbyte/s) were stored locally or on a data server for further offline analysis. Spike detection analysis was performed by employing the Precise Timing Spike Detection (PTSD) algorithm (Maccione et al., 2009) integrated in the Brainwave software application (3Brain GmbH, Switzerland). A threshold of 9 times the standard deviation was used for spike detection, whereas burst events were identified if at least five consecutive spikes in an Inter Spike Interval (ISI) lower than 100 ms were detected. In our analysis, we considered only electrodes recording spikes with rates between 0.05 and 10 events/sec, i.e., “active electrode.” All spike trains were exported from BrainWave to Matlab (The MathWorks Inc., USA) files and were analyzed with custom Python scripts (Python Software Foundation), in particular for first order statistics of network-wide mean activity parameters, i.e., mean firing rate, active electrodes, and for the analysis of the neuronal firing frequencies distributions, where lognormal-like distributions and fit statistics were done as described in Amin et al. (2015).

### Multi-site electrical stimulation on CMOS-MEAs and analysis of evoked responses

The hiPSC-derived neuronal networks were electrically stimulated with the on-chip integrated stimulating electrodes of the CMOS-MEAs chip. In our custom experimental platform, the 16 stimulation electrodes were connected to a 16-channel electrical stimulator (Plex-Stim Electrical Stimulator 2.0 System, Plexon Inc., USA) controlled with a custom software tool developed in C# programming language. Low-frequency (0.2 Hz) trains of biphasic current stimuli (300 μs per phase, positive phase first, peak-to-peak amplitude of 300 μA) were delivered for 3 min to all the 16 stimulation electrodes using a random sequence for the stimulation sites applying each stimuli, i.e., providing 35 stimuli for each electrode channel. As for spontaneous activity recordings, the 4096 recorded extracellular signals were exported and analyzed with custom Python scripts (Python Software Foundation). The electrically evoked responses were isolated from the recordings by binning the overall network spiking activity with a bin size of 25 ms. The detection of the stimulation artifacts was afforded with a hard threshold (Dayan and Abbott, 2005). Afterwards, the evoked responses corresponding to 35 repetition of a stimulus for each site were aligned to the time zero and binned with 25 ms bin size. The post-stimulus-time-histograms (PSTH) (Gerstein and Kiang, 1960) was computed from these isolated and aligned responses. The first 10 ms after the stimulus were discarded in order to avoid any possible effect of the stimulus artifact. The PSTHs were computed for each stimulating electrode by computing the average, the lower and uppermost percentiles over all evoked activities for each time bin. Additionally, to characterize the two forms of network evoked responses, i.e., fast and long-lasting, we computed the timing of first occurring spikes. First, globally, we accumulated statistics on latencies of first spikes in response to each train of 35 stimuli from all stimulating sites. Secondly, this approach was extended to test the effect of the distance from the stimulating site on the first spike occurrence in 500 ms time window by considering a circular region spreads from the close proximity of the stimulus, i.e., 4 electrodes (~50 μm), to the distant ones, i.e., 30 electrodes (~2827 μm), spacing 2 electrodes (~12.5 μm). Responses were considered reliable for computation when electrodes provided responses to at least 10 out of 35 low-frequency stimulation trials.

### Analysis of spontaneous and evoked spike waveforms

Spike waveforms were analyzed in a similar way as previously reported method (Nam and Wheeler, 2011) and based on a subset of parameters characterizing the spike shape. Also for this analysis, the spike detection was performed with the BrainWave acquisition software using the PTSD algorithm. The artifact detection was applied for these electrically evoked datasets. The spike waveforms were exported as Matlab files (time windows of 4 ms, centered on the peak of the spike) and, in order to sort the spike waveforms with the Offline Sorter™ (Plexon Inc., USA), these files were converted into.nex files with a custom Matlab® script (MathWorks, USA). Spike sorting was performed with a t-distributions expectation-maximization (T-dist E-M) algorithm (Shoham et al., 2003) including a unit range of 1–3.

### Immunofluorescence protocol

Culture medium was removed and cell culture washed using phosphate-buffered saline (PBS) at 37°C, then cells were fixed in paraformaldehyde (4% in PBS-1X) for 15 min at room temperature (RT). Following fixation, cells were washed gently 4 times with PBS-1X and then cells were permeabilized with 0.1% Triton in PBS-1X (PBST) for 10–15 min and blocked with 5% Normal Goat Serum (NGS) (EuroClone S.p.A, Italy) for 1 h before incubation with primary antibodies. Cells on CMOS-chips were then incubated overnight at 4°C in primary antibodies diluted in the blocking buffer. The following primary antibodies were used; guinea pig anti-MAP2 (1:1000), mouse anti-Synaptophysin-1 (1:200), rabbit anti-V-GAT (1:500), guinea pig anti-V-GLUT 1 (1:1000), mouse anti-GAD65 (1:500) (all from Synaptic Systems, Germany), as well as rabbit anti-c-Fos (1:500) (Calbiochem Millipore, Italy), and mouse anti-PSD-95 (Thermo Scientific, Italy). Progressive 3 washing steps in PBST for 5 min per wash were performed, and cells were incubated for 1 h in the dark at RT with the corresponding secondary antibodies including; Alexa Fluor 488, Alexa Fluor 546, and Alexa Fluor 647 (all from Life Technologies, Italy). Afterward, cells were washed 4 times with PBST and incubated for 15 min in the dark at RT with the nuclear marker Hoechst 33342 (1:500) diluted in PBS-1X (ThermoFisher Scientific, Italy). Images from iCell neurons were acquired and visualized with 20X and 40X objective lenses using Leica SP5 upright confocal microscope (Leica Microsystems, Germany), Nikon A1 inverted confocal microscope (Nikon Instruments, Italy), and a BX61 Olympus fluorescence microscope (Olympus Life Science Solutions, Italy).

### Image analysis

**Figure 2:**
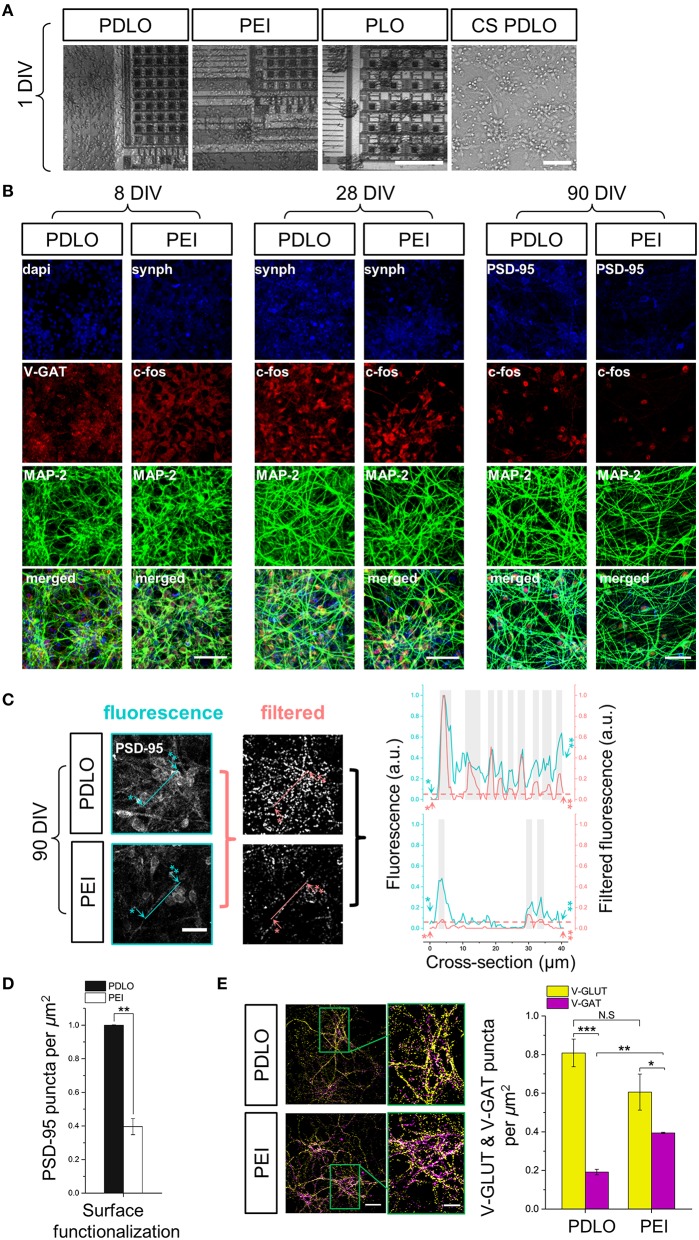
**Optical read-outs allow to track the development of human iCell neuronal networks over 3-month culture and to discriminate the effects of biochemical adhesion molecules on neuronal growth and function. (A) Fluorescence images elucidate differences in neuronal growth after 1 DIV on** CMOS-MEAs and glass coverslips coated with PDLO, PEI, and PLO adhesion molecules. Cultures grow homogenously on PDLO and PEI, but they cluster and fascicule on PLO surfaces. Scale bars represent 100 and 50 μm, from left to right, respectively. (**B**) Confocal micrographs of iCell neurons at 8, 28, and 90 DIV show expression of typical neuronal and synaptic proteins, i.e., Synaptophysin-1, GAD-65, and PSD-95 (blue), V-GAT, and c-FOS (red), in addition to MAP-2 (green). At 28 DIV, neurons grow on PDLO, and PEI show no distinguishable differences between their cellular neuronal activity (c-FOS) and presynaptic expression (synaptophysin-1), whereas at 90 DIV, the expression of post-synaptic protein PSD-95 is prominently higher in cultures grown on PDLO than others grown on PEI, which provides evidence of different maturation profiles. Scale bars represent 50 μm for (8, and 28 DIV), and 30 μm for 90 DIV. (**C**) Quantification of PSD-95 expression. To quantify the puncta of PSD-95 constructs, images were processed with the granulometric filtering (see Materials and Methods and Supplementary Figure 2). Cross-sections elucidate the intensity and density of PSD-95 constructs. Blue and red cross-sections depict the fluorescence and the corresponding filtered intensities, respectively. Single and double asterisks illustrate the starting position of the cross-section, i.e., 0 μm, and its end, i.e., 40 μm, respectively. The position of PSD-95 puncta was defined above an arbitrary offset, i.e., 0.07, here indicated by gray bars. Scale bar represents 20 μm. (**D**) Bar graph showing the quantified effect of surface functionalization on the expression of PSD-95 ensembles (density). PSD-95 level significantly reduced 2.5-fold in neurons grown on PEI compared with neurons grown on PDLO-coated substrate, (*n* = 3, at least 9 image fields per sample, ^**^p < 0.01). (**E**) At 90 DIV, quantifications performed on confocal micrographs (*n* = 3, at least 9 image fields per sample, ^**^p < 0.01, ^***^p < 0.001) illustrate lower expression of V-GAT in neurons grown on PDLO than those grown on PEI. Scale bars represent 50 μm (left), and 20 μm (right) for the magnified region. N.S, indicates non-significant.

In order to quantify the PSD-95 assembles in various densities of human neuronal network coupled to CMOS-chip, we performed the granulometric filtering (Prodanov et al., 2006; van den Bogaart et al., 2011) method on the confocal micrographs. Images were converted to greyscale to enhance the contrast between signal and noise, followed by setting a manual threshold. Afterward, small grains of PSD-95 construct, consisting of several pixels, were identified based upon their sizes and shapes in a heterogeneous background image. Integral intensity thresholding then was used to assign the granulometric filtering intensities and performed using Imagej analysis software platform (Schneider et al., 2012). The blue and red cross-sections display the fluorescence and the corresponding filtered intensities, respectively (see Figure 2C and Supplementary Figure 2). Arbitrary offset, i.e., dashed line at 0.07, was defined to pinpoint the PSD-95 puncta above that level while the yellow bars represent the location of the puncta above the offset. On the other hand, PSD-95 expression construct, V-GLUT, and V-GAT densities were performed using automatic particle counting integrated in Imagej platform. The synaptic density was hence analyzed by counting immunoreactive puncta per μm^2^ in at least 9 visual fields, randomly chosen in multiple cultures (*n* = 3), measuring ~ 79 × 79 μm from the 1024 × 1024 pixel image, where only fluorescence puncta that had their major axes within 0.3–1.3 μm were selected for analysis (Ziff, 1997).

### Statistical analysis

All data are expressed as the mean ± standard error of the mean (SEM). Differences between groups were examined for statistical significance, where appropriate using one-way analysis of variance (ANOVA) or two-way ANOVA followed by Tukey *post-hoc* testing. *P* < 0.05 was considered significant. Furthermore, a non-parametric Kruskal-Wallis method was used for data not normally distributed.

## Results

### Optical read-outs reveal synaptic formation and spontaneous activity during development of human iCell neuronal networks

Human-derived networks of iCell Neurons were grown on CMOS-MEAs up to 3 months. In order to optimize the cell culture protocol, different adhesion-promoting molecules were tested by characterizing the cell morphology and network formation over development (see Materials and Methods).

After 24 h, cells adhered on all coated substrates and started extending neuritic processes, as shown in Figure 2A. Indeed, iCell neurons featured homogenous growth on PDLO and PEI substrates even after 1 day *in vitro* (DIV); while neurons grown on PLO substrates were more clustered, with fasciculated processes (see Supplementary Figure 1).

Next, to investigate the induced effects of the substrate conditions on the growth and iCell neuronal function, confocal micrographs of immunostained cultures were obtained over network development, for 3 time sets, i.e., at 8, 28, and at 90 DIV. As shown in Figure 2B, synaptic formation of iCell neuronal network was examined by the quantification of expression of the major synaptic vesicle membrane proteins, i.e., synaptophysine-1, as well as markers for post-mitotic neural subtypes, composed by the inhibitory synapses, i.e., GABA transporter in the membrane of synaptic vesicles (V-GAT) and synthesizing GABA, the major inhibitory neurotransmitter in the CNS (GAD-65), and excitatory synapses, i.e., Glutamate transporter in the membrane of synaptic vesicles (V-GLUT),. Such a heterogeneity in the expression of synaptic neuronal markers is important to ensure physiological functional activity and it might be given as an intrinsic property of these human-derived neuronal cells that likely underpins their functional diversity among cultures (Llinas, 1988). The major microtubule associated protein was visualized with the MAP-2 marker and its expression showed a salient healthy neurite growth during developmental phases. Additionally, confocal optical read-outs of c-Fos expression were used to quantify spontaneous activity in these cultures at cellular resolution for specific time sets. The expression of neuronal c-Fos marker suggested that cellular spontaneous neuronal activity can be detected already from 8 DIV and up to the entire experimental window of 90 DIV.

We then specifically investigated the effects of PDLO with respect to PEI on the maturation and stability profile of synapses in these human-derived neuronal networks. For this purpose, at 90 DIV, we performed immunostaining to assess the expression levels of PSD-95 in iCell neurons grown on CMOS-MEAs coated with these two molecules as shown in Figure 2B. Notably, the fluorescent puncta of PSD-95 were significantly reduced 2.5-fold in case of neurons grown on PEI coated-surfaces compared to those grown on PDLO surfaces, as illustrated in Figures 2C,D, and also confirmed with low density human-derived neuronal cultures in Supplementary Figure 2. Since PSD-95 proteins have been reported to play a central role in balancing the ratio of excitatory/inhibitory synapses (Prange et al., 2004), we also investigated the ratio of counted V-GLUT/V-GAT positive puncta obtained from iCell neurons at 90 DIV. Interestingly, Figure 2E and Supplementary Figure 3 shows that this ratio is (4.2 ± 0.18)-fold for neurons seeded on PDLO substrates, whereas it is (1.53 ± 0.08)-fold reduced significantly for cultures grown on PEI. This indicates a salient difference in inhibitory synapses for networks grown on the two coatings that resulted from the significantly higher expression of V-GAT that leads to an imbalance of the ratio of glutamatergic/GABAergic synapses for networks grown on PEI-coated substrates rather than those grown on PDLO-coated substrate. In turn, this provides evidence that PDLO is a very suitable surface functionalization for establishing a functional human derived neuronal network, while maintaining their physiological maturation and synaptic functional plasticity.

### Spontaneous electrical activity of human iPS derived neuronal networks undergoes developmental changes over 3 months of growth

#### Developmental changes described by network-wide activity parameters

**Figure 3:**
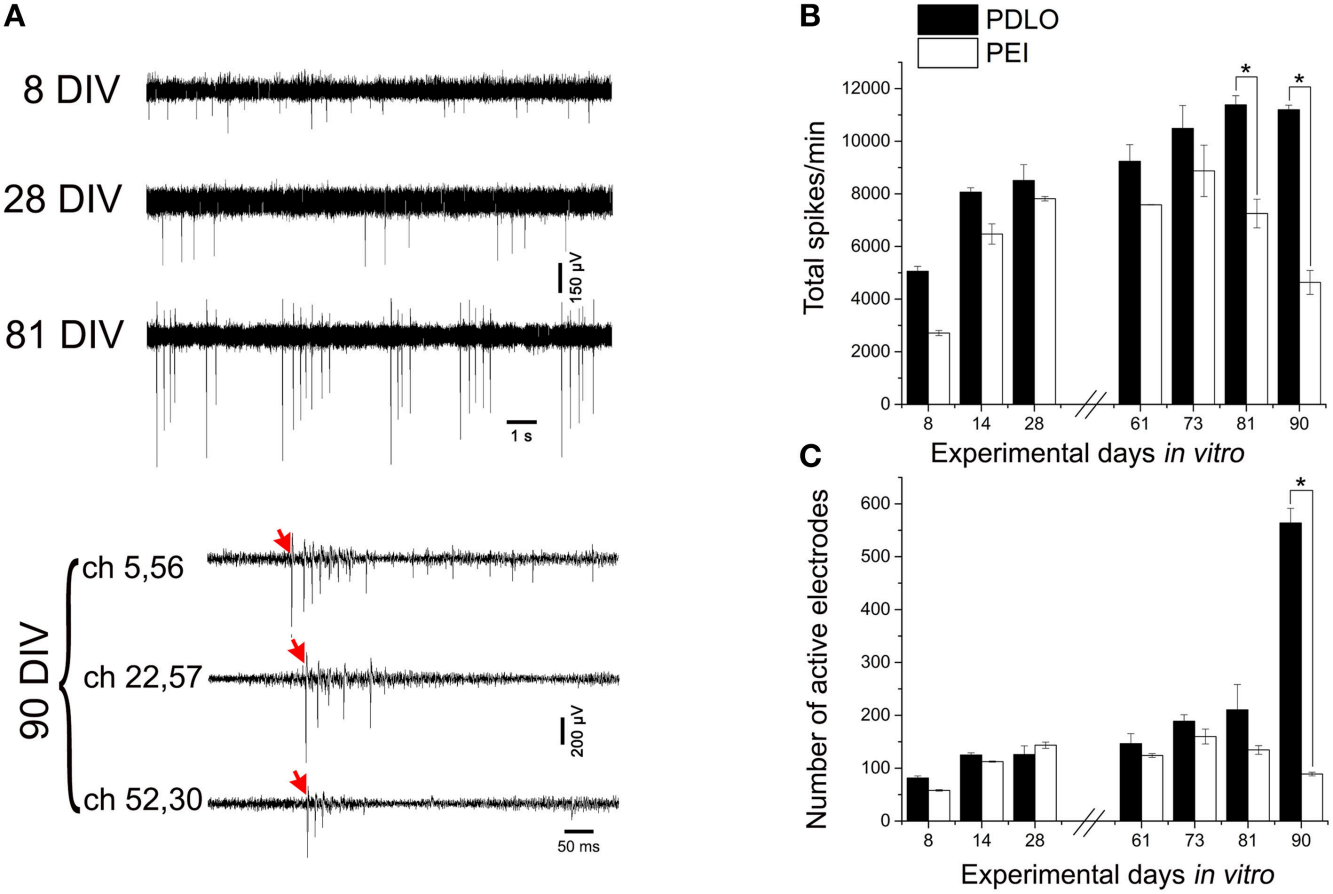
**High-resolution electrical read-outs with CMOS-MEAs unveil changes in spiking activity of iCell neuronal networks during development**. (A)Typically recorded extracellular signal traces associated with the detected firing rates during development. The activity develops from single spikes (8 DIV), tonic firing (28 DIV) to bursting, and synchronized spikes (81, and 90 DIV). Red arrows indicate start of propagating burst. **(B)** Bar graph showing the effect of surface functionalization on the evolution of network-wide spiking activity, i.e., total spontaneous spikes, per 10 min recording phases. Spontaneous firing rates of cultures grown on PDLO coated substrates tend to gradually increase during development to reach a peak at 81 DIV, followed by a plateau at 91 DIV. A similar initial trend (lower rate) is observed for cultures grown on PEI coated substrates cultures, with a peak at 73 DIV (earlier than PDLO cultures). Afterward, firing activity significantly declines at 81, and 90 DIV (*n* = 3 for each substrate, ^*^p < 0.05, one-way ANOVA). Significant differences of firing activity measures between substrate (PDLO vs. PEI) and DIV (8, 14, 28, 61, 73, 81, and 90) were determined by two-way ANOVA followed by Tukey's *post-hoc* test (^*^p < 0.05). **(C)** Bar graph illustrating the dynamic changes in the total number of active electrodes over 3 months of culture, on PDLO and PEI coated substrates. Consistently with the development of the spiking activity shown in **(B)**, the number of active electrodes indicates an increasing tendency. A higher increase is observed for cultures grown on PDLO than PEI and this tendency becomes significantly higher for cultures on PDLO at 81, and 90 DIV (*n* = 3 for each substrate, ^*^p < 0.05, one-way ANOVA). Significant differences of detected active electrodes between substrate (PDLO vs. PEI) and DIV (8, 14, 28, 61, 73, 81, and 90) were determined by two-way ANOVA followed by Tukey's *post-hoc* test (^*^p < 0.05).

The CMOS-MEA chips enable simultaneous extracellular neuronal recordings from a dense array of 4096 electrodes (see Materials and Methods), thus providing high-resolution recordings of spiking activity all over the network, as required for a precise quantification of mean activity parameters that characterize network-wide firing patterns over development (see Figure 1). In order to assess whether surface functionalization might induce different electrophysiological behaviors over network development, we evaluated the spontaneous activity over a time window ranging from 8 DIV and up to 90 DIV. Figure 3A shows some typical signal traces of the firing patterns recorded at different time-points over the 3 months of cell culture, while total detected spontaneous spikes, per 10 min recording phase, and the number of electrodes recording spiking activity, i.e., active electrodes, are reported in Figures 3B,C. It can be observed that there is an oscillatory behavior in the spiking profile and a shift from random spiking activity that typically characterizes young cultures; to tonic network-wide spiking, manifesting bursting events at 81 DIV and stable burst firing patterns for networks at 90 DIV (see Supplementary Figure 4). These developmental changes in the spontaneous activity suggest that the iCell neuronal network undergoes maturation, similarly as what has been reported for murine primary cultures. However, the development of murine neurons requires much shorter time-window for their development, i.e., a few weeks, of network-wide activity (Chiappalone et al., 2006; Wagenaar et al., 2006). The developmental changes in the network-wide spiking activity mirror the evolution in the number of active electrodes that is preserved throughout the different stages of development, with the exception of a considerable sharp increase at 90 DIV for cultures grown on PDLO. This increase is not observed for networks grown on PEI. Indeed, our results show that iCell neurons begin firing action potentials at an early age (8 DIV), a time that extracellular recordings are mainly composed by signals with single random spikes, with a fairly low number of active electrodes. At 81 DIV, the spiking activity substantially increases to reach a peak value and a plateau at 90 DIV for PDLO cultures; whereas for PEI cultures, the peak is reached earlier at 73 DIV and it is followed by a significant decay in the firing rate at 90 DIV. It is important to note that neurons grown on PLO coated substrates did not exhibit any detectable extracellular spikes, notwithstanding the cellular activity indicated by c-Fos expression as shown in Supplementary Figure 1. This might be resulting from the clustered morphology of cultures grown on PLO-coated substrates, which impairs the neuron-electrode coupling by a weak cellular adhesion to the substrate. Thus, given the lack of activity and the clustered morphology, cultures grown on PLO were washed-out after 3 weeks.

#### Developmental changes unveiled by the distributions of the neuronal firing frequencies

The distribution of the iCell neuronal firing frequencies in the network was computed from the spontaneous extracellular signals simultaneously recorded at sub-millisecond resolution form the 4096 electrodes of the CMOS-MEA. This distribution can reveal the frequency contributions of all the differently spiking neurons in the developing network. Thus, it can be an indicator of the overall functional changes occurring at the cellular scale and goes beyond the solely monitoring of network-wide activity parameters by revealing changes occurring over time to both low-firing and high-firing neuronal populations that constitute the network.

**Figure 4:**
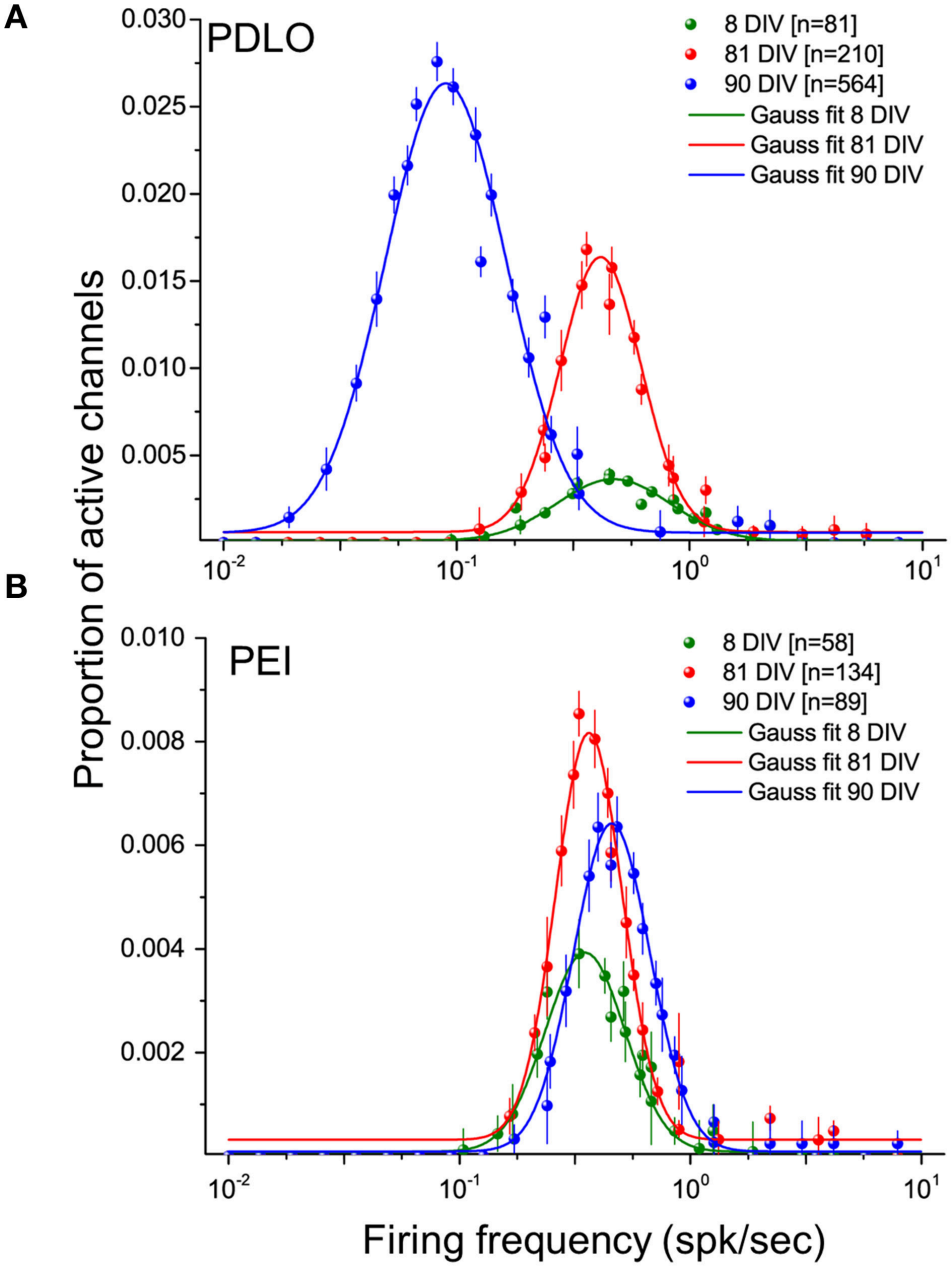
**Distributions of the firing frequencies of iCell neuronal networks is lognormal- like and allow to elucidate the dynamic behavior of human iCell neuronal networks during development.(A)** Gaussian fits of the firing frequency distributions for three developmental phases of neurons grown on PDLO coated substrates, i.e., at 8, 81, 90 DIV. The means of the distributions at 8 and 81 DIV hold the same frequency position, but a higher peak of the distribution (red) is observed at 81 DIV. At 90 DIV, the mean of the distribution (blue) shifts left toward low-firing rates and the occurrence of active electrodes reaches its peak at this range of low frequency. **(B)** Neuronal cultures grown on PEI coated substrates hold over development the means of their lognormal-like distributions at the same frequency range (no shift). The peak of these distributions indicates a lower probability of active electrode occurrence during development compared with networks frown on PDLO coated substrates.

As reported in Figure 4, our large-scale electrode array recordings show that iCell neuronal networks exhibit a lognormal-like distribution of neuronal firing frequencies, similarly as what has been reported for *in vivo* recordings on rodents, monkeys and human (Buzsáki and Mizuseki, 2014) and, in our previous work, for *in vitro* murine neuronal networks (Amin et al., 2015). In order to quantitatively determine changes in the distribution occurring during network development, here we have computed the distribution for different time points, i.e., at 8, 81, and at 90 DIV, and we have used the mean parameter of the Gaussian fit. As shown in Figure 4A and Supplementary Figure 5A, when neurons grow on PDLO substrates, the mean parameter suggests that the distributions at 8 and 81 DIV tend to be nearly the same. This is followed by a significant 5-fold shift toward low firing rates at 90 DIV and an increase in the peak of the distribution. On the other hand, for networks grown on PEI substrates, the mean parameter of the Gaussian fit of spiking frequency distributions at 8, 81, and 90 DIV shows a tendency to maintain the same distribution with almost the same peak value, as in Figure 4B and Supplementary Figure 5B.

### Human iCell neuronal networks can respond to electrical stimulation after 3 months of development

#### Human iPS derived neuronal networks respond to electrical stimulation

**Figure 5:**
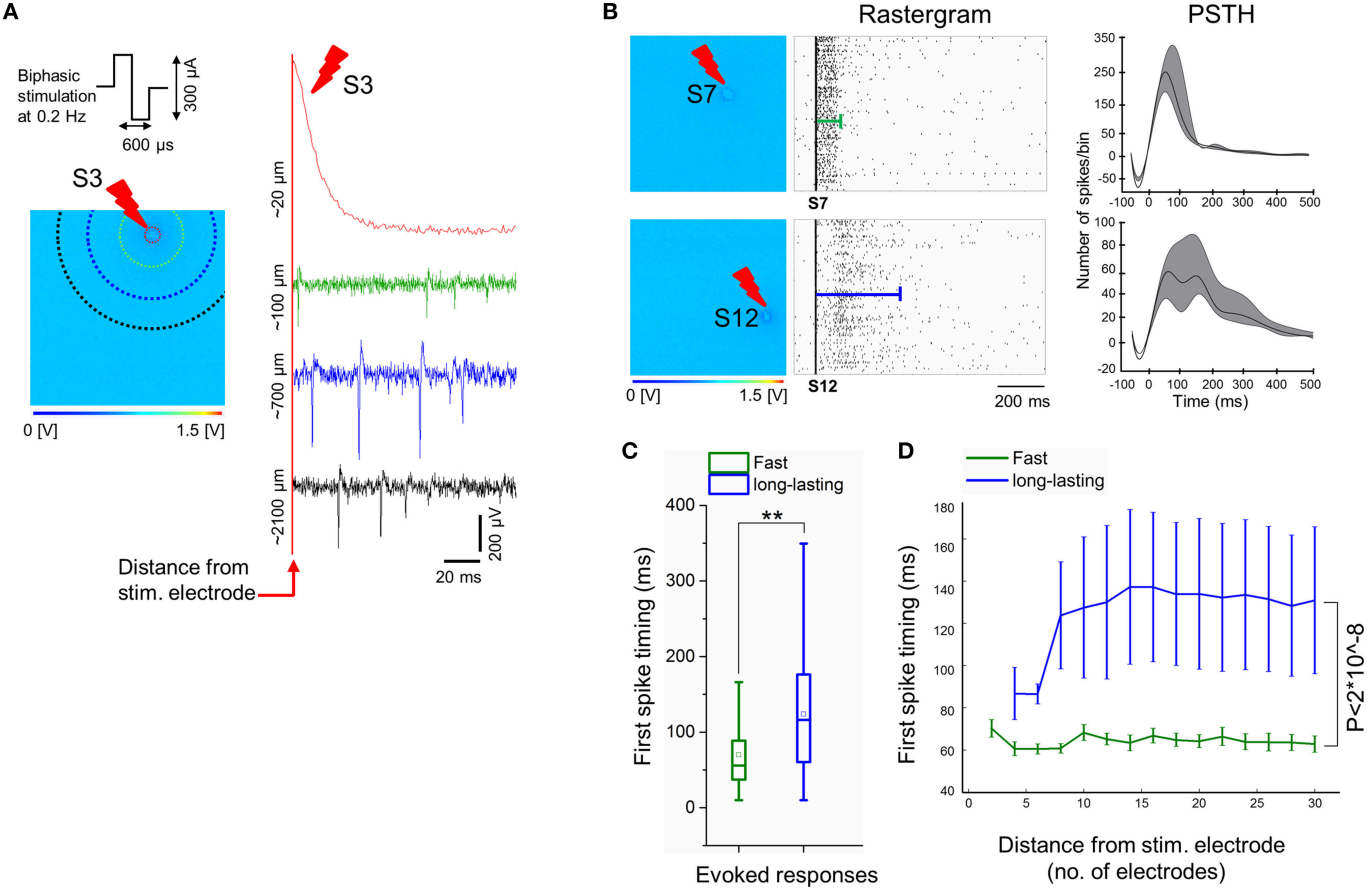
**Electrical stimulation delivered by electrodes on-chip show evoked responses only at 90 DIV. (A)** Sample of evoked signal traces illustrates the confined stimulation artifact to electrodes close to the stimulating electrodes (< 100 μm), e.g., when low-frequency (0.2 Hz) trains of biphasic current stimuli (300 μs per phase, peak-to-peak amplitude of 300 μA) is delivered for 3 min to exemplary electrode S3. Colors of signal traces correspond to the distance from the stimulating site, i.e., correlated with colors of dotted circles in the 64 × 64 array; for instance, the green response occurred at ~6 electrodes distance (~100 μm) from the stimulating site, while blue at ~15 electrode distance (~700 μm), and black trace at ~26 electrode distance (~2100 μm). **(B)** Exemplary electrical stimuli (S7 and S12) delivered by specific electrodes on the 4096 electrode arrays (left) and their corresponding evoked responses by means of rastergram and PSTH analyses (middle and right, respectively). Two forms of evoked responses were observed and show, fast responses lasting < 100 ms (top), and long-lasting responses up to ~500 ms (bottom). In the middle, rastergrams showing a sequence of evoked spikes that are confined within the green line (top), while evoked spikes are spread along the blue line (bottom). On the right, exemplary PSTHs that are computed from all evoked activities corresponding to their stimulating electrodes (see Materials and Methods). This shows that fast responses are characterized by a higher number of spikes per 25 ms bin size and lasting < 100 ms, in contrast with a lower number of spikes per bin and duration up to ~500 ms in case of long-lasting responses. Note that the negative tough in the PSTH is determined by the adopted artifact removal and smoothing procedures. **(C)** Average of the first spike timing delays with respect to electrical stimulation computed from two-group responses (fast vs. long-lasting). A delay of about 70 ms is observed from fast responses (green), while the onset of long-lasting responses (blue) occurred significantly over a longer period, i.e., 120 ms (*n* = 3, ^**^p < 0.01). **(D)** The average of first spike timing is measured with respect to the distance from the stimulation site, by starting from four electrodes (~50 μm) until 30 electrodes (~2827 μm) after stimulus. This indicates that the first spikes in case of fast responses (green) occur within ~60–70 ms after stimulation, regardless of their distance from the stimulus, whilst, first spikes in long-lasting responses (blue) begins at ~85 ms, in close proximity to the stimulus (within four electrodes distance), and delayed to 120–140 ms in the distant ones. Non-significant differences of first spike timing between response (fast vs. long-lasting) and distance in μm (5, 10, 15, 20, 25, and 30) were determined by two-way ANOVA (*p* > 0.05). Responses of both groups (fast vs. long-lasting) are highly significant over the entire computed distance, i.e., ~2827 μm. (*n* = 3, *p* < 2^*^10^∧−8^, two-way ANOVA).

For different ages of the iCell neuronal culture, trains of low-frequency electrical stimuli at 0.2 Hz were applied sequentially from the 16 stimulation electrodes integrated on the CMOS-MEAs. These electrodes are dedicated to electrical stimulation and are interleaved within the 4096 recording electrode array. In addition, pre-stimulation and post-stimulation spontaneous network activities were recorded. We have observed that only neurons grown on PDLO coated substrates and exclusively after 90 DIV were capable to elicit spiking responses to electrical stimuli. Samples of evoked signal traces are shown in Figure 5A. Interestingly, due to the electrode embedded morphology, the stimulation artifact is confined to < 20 μm distance from the stimulation electrode, thus allowing to record spike evoked responses within 7 ms latency after stimulation, i.e., < 100 μm, and to observe propagating responses in the network. Under our cell culture conditions, these responses consist in different spatiotemporal patterns of spikes that appeared depending from the different stimulation sites (see Supplementary Movie 1). Our results show two major forms of electrically evoked network responses that can be distinguished from the rastergrams and from the post-stimulus-time-histograms (PSTHs) as reported in Figure 5B. A first form consists in fast spiking responses of the network lasting for ~100 ms after the stimulation (e.g., stimulation electrode S7) followed by a network activity that returns to the pre-stimulation activity baseline. These responses are similar to those observed in murine primary neuronal cultures (Jimbo and Kawana, 1992). A second form of responses was observed for stimuli delivered from some electrodes (e.g., stimulation electrode S12) and consisted in long-lasting spiking responses up to ~500 ms in delay, after the stimulation instant. To the best of our knowledge, this second form of evoked responses has not been observed in primary murine neuronal cultures and might be the result of either the cell-type heterogeneity of developing networks or of an intrinsic property of these human iCell neurons.

To further characterize the evoked spiking responses, we have also investigated the delays of the first evoked spikes after stimulation, by taking into account the two forms of responses, i.e., fast and long-lasting. The Figure 5C shows an overall average of the first spike latency for stimuli delivered by different stimulation sites, as recorded regardless of the distances of the recording electrodes. First, spike latencies were significantly lasting shorter than ~70 ms for fast responses and ~120 ms for long-lasting responses. Secondly, we have tested the relationship between first spike latency and the distance from the stimulating electrode, by considering that this distance can be maximally of ~2827 μm in a radius of 30 electrodes (see Figure 5D and Materials and Methods section). This analysis reveals that the first spike timing in two forms of evoked responses, i.e., fast and long-lasting, did not consistently depend on distance from the stimulating electrode, though the first spike timing of long-lasting responses was significantly delayed compared to fast responses along ~2827 μm distance from the stimulating electrode.

Finally in order to evaluate whether low-frequency stimulation affects the iCell network activity, the number of active electrodes and the mean firing rate were computed for pre- and post-stimulation recordings. The number of active electrodes increased slightly, i.e., from 564 ± 28 to 688 ± 21, before and after stimulation, respectively. This is accompanied by a slight decrease in the mean firing rate (spk/s), i.e., from 0.66 ± 0.03 to 0.58 ± 0.03, before and after stimulation, respectively. Notably, despite these slight changes, no evidence for clear changes induced by the electrical stimuli occurred in the network features, thus suggesting no considerable plasticity being induced by low-frequency stimuli into these human derived neuronal networks.

#### Repertoire of physiological extracellular spike signals unveiled upon electrical stimuli

**Figure 6:**
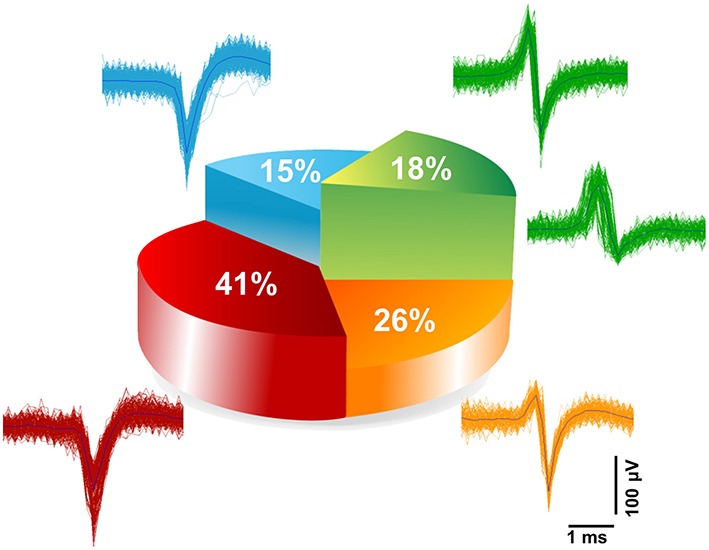
**Categorization of extracellular spike waveforms of iCell neuronal recorded**. Waveform classifications of evoked responses detected and sorted at 7.8 kHz sampling frequency. Two main classes were found; belonging to negative spikes namely (monophasic 41%, biphasic 15%, and triphasic 26%), and positive biphasic spikes (18%).

The electrically evoked spike waveforms were analyzed in order to describe the repertoire of extracellular spike waveforms of iCell neuronal networks and for comparison with those reported for primary neuronal cultures from rat or mouse embryos. To do so, we have used a spike sorting algorithm included in the Offline Sorter (from Plexon Inc., USA) (see Materials and Methods). Given the relatively low sampling frequency, i.e., 7.8 kHz/electrode, of our electrode signals when CMOS-MEAs are operated to record from the whole array of 4096 electrodes, we have at first assessed the sorting performances. To do so, we have compared spike sorting results obtained from the same culture and the same electrode area with extracellular signals sampled at the normal frequency, i.e., 7.8 kHz/electrode, and at a higher frequency of 22 kHz/electrode. This was made possible since CMOS-MEAs act as imaging sensors that allow to select a region of interest, i.e., an area of the electrode array, and thus to increase the sampling frequency in this recording modality. Details on the approach are reported in the Materials and Methods. It has to be highlighted that high sampling frequencies are particularly important for spike sorting *in vivo* recorded extracellular signals, but might not be needed for cell culture signals given the much lower cellular density and bi-dimensional organization of the network. This consideration was confirmed by our results. Indeed, the spike sorting results for the same network area and for both sampling frequencies do not show significant differences in the number of sorted units and in the features of the spike shapes. In particular, results show that most of the electrodes record single-units (>95% [*n* = 3]) and only a very few electrodes record two units (< 5% [*n* = 3]), (see Supplementary Figure 6). Following this, we have categorized the repertoire of spike waveforms expressed by iCell neurons upon recording at 7.8 kHz/electrode from the whole array of 4096 electrodes, as shown in Figure 6. As reported for embryonic neuronal cultures from rats (Nam and Wheeler, 2011), categorization of the waveforms was done based on the amplitudes of the positive and negative peaks of the sorted spikes. Similarly as in the Nam and Wheeler paper for murine cultures, we found that the majority of the evoked spike waveforms (82%) are negative spikes, i.e., featuring a larger amplitude for the negative peak, while only a small fraction of spikes are positive (18%), i.e., with a larger amplitude for the positive peak. Further categorization indicates that 41% of the spikes are monophasic negative events, 15% are biphasic negative spikes, and 26% are triphasic negative spikes characterized by a small amplitude positive peak that is followed by a large amplitude negative peak and by a third small amplitude positive peak. The positive spikes were only biphasic waveforms, with a large amplitude positive peak followed by a negative peak. Interestingly, these results on sorted spike waveforms show that human neuronal cultures exhibit very similar extracellular waveforms as reported for physiological extracellular signals observed for embryonic primary neuronal cultures from mice or rats.

## Discussion

Human-derived neurons represent an emerging opportunity to accelerate research on brain diseases. However, to the best of our knowledge, the electrophysiological properties of networks formed by these human pluripotent stem cells (hiPSC) derived neurons were only partially characterized and cell culture protocols for their growth and maintenance need to be optimized for electrical read-outs.

As known from several decades of research on neuronal networks prepared from rodents, viable networks should express both developing patterns of spiking activity during their growth *in vitro* and responsiveness to electrical stimuli consisting in network-wide elicited spiking activity (Jimbo and Kawana, 1992). Here, upon optimization of the cell culture conditions and by applying a high-resolution CMOS-MEA with 4096 recording electrodes and 16 stimulating sites, we show that networks of a commercially available line of hiPSC–derived neurons can indeed express all these network-wide electrophysiological properties when grown for 3 months *in vitro*. Differently than previously reported results (Odawara et al., 2014), throughout the combined use of fluorescence imaging and electrical recordings, our results also show that astrocyte-neuron co-cultures are not mandatory for network development. Rather, an optimized surface functionalization utilizing PDLO is found to be a prerequisite. In the next sections we shortly discuss the major findings of this work.

### Poly-dl-Ornithine coated surfaces promote morphological and functional development of human derived neuronal networks

The morphological and functional effects induced by three different biochemically active molecules, i.e., PDLO, PEI, and PLO, deposited on the substrate surfaces before cellular seeding were experimentally investigated with confocal microscopy and CMOS-MEA recordings, up to 3 months of cell culture. In particular, we have evaluated the ability of these three substrate functionalization to support cellular attachment, long-term culture viability and to enhance neuroelectronic coupling. Similar results were obtained both on glass coverslips and CMOS-MEA substrates, thus discarding possible effects induced by the different surface topologies of the substrates.

Morphologically, although differences have already been qualitatively noticed after 1 DIV, surfaces that were coated with PDLO and PEI showed homogeneous cellular growth and maintained distributed distinct cells forming the network. However, cultures grown on PLO coated substrates showed a strong prominence to the formation of cellular clusters and fasciculation of the neuritic processes. Such morphological differences among cultures grown on differently coated substrates were also reflected in our electrical measures. Indeed, networks grown on PLO coated CMOS-MEAs did not show spontaneous activity and neither evoked responses, despite the high-electrode density that is expected to allow recording also in case of a very few spiking neurons. On the other side, spontaneous electrical activity was recorded from CMOS-MEAs coated with PDLO or PEI, with significant differences induced by the two coatings during network development.

Evoked responses to electrical stimulation were recorded only from PDLO-coated devices. Thus, our results indicate that this bioactive molecule seems to be better suited to grow networks of hiPSC derived neurons. A reason of this particular feature of the PDLO might be conferred by, first, the additional carbonyl groups which contribute negative charges, and secondly, to the structure of ornithine molecules built up in a high molecular weight of the polycationic organic substance, thus providing sufficient binding sites for cell attachment. Given the lack of responsiveness to electrical stimulation of cultures grown on PEI-coated substrates, we examined the formation and stabilization of iCell neuronal synapses in comparison with responsive cultures grown on PDLO-coated substrates. Since synaptic transmission prevails the cellular communication between neurons and it is required for brain function; hence, stabilization of synapses and orchestration of the pre- and post-synaptic terminals are critical to synaptic development, plasticity and thus neuronal dynamics, as conferred by hardwired neural networks.

Throughout the quantification of the expression level PSD-95 (see Materials and Methods), i.e., a membrane-associated protein predominant in the post-synaptic density that is implicated in maturation, synaptic strengthening, and plasticity, we show that PSD-95 is highly expressed in neurons grown for 3 months on PDLO coated substrates, while it is significantly lower for neurons grown on the PEI coated ones. Since PSD-95 promotes the stabilization of young synapses (Taft and Turrigiano, 2014), our data suggest a developmental regulated process that results in more mature and stable young synaptic contacts for neurons grown on PDLO surfaces compared to neurons grown on PEI substrates.

Previously reported evidences converge on the fact that appropriate expression of PSD-95 enhances synaptic clustering of α-amino-3-hydroxy-5-methyl-4-isoxazole propionic acid receptor (AMPAR), i.e., GluR1 subunit, that in turn increases excitatory presynaptic input (El-Husseini et al., 2000) as observed in our data by V-GLUT quantifications.

Interestingly, by assessing the balance between excitatory and inhibitory synapses, as indicated by the V-GLUT/V-GAT ratio at 90 DIVs for both networks grown on PDLO and PEI coated substrates, we have found significant differences in the neuronal excitability (Prange et al., 2004) of the two types of networks. These data suggest an effect by which the ratio of excitatory to inhibitory synapses (V-GLUT/V-GAT) is lower in neuronal networks grown on PEI coated substrates (i.e., ~60% V-GLUT and ~40% V-GAT), due to an increase in inhibitory synapses and a decrease in excitatory synapses, possibly favoring inhibition, compared with a higher ratio for neurons grown on PDLO coated substrates, i.e., ~80% V-GLUT and ~20% V-GAT, thus developing with an excitation preference and prone these networks to respond to electrical stimuli. It should also be noted that our quantification of excitation and inhibition is inline with data reported for neurons in the cerebral cortex, where ~80% are excitatory glutamatergic neurons and ~20% inhibitory GABAergic interneurons (Wonders and Anderson, 2006). The mechanisms by which PEI induces such a network development with tendency to inhibition is unclear and they might be associated with other scaffolding proteins and cell adhesion molecules (Levinson and El-Husseini, 2005).

### Human-derived neuronal networks grown on PDLO and PEI-coated substrates express different spontaneous activity patterns and show lognormal-like distributions of their neuronal firing frequencies

High-resolution CMOS-MEA recordings from 8 to 90 DIV, show that in an early stage of network formation of hiPSC–derived neurons grown on PDLO-coated substrates, the network-wide firing patterns are characterized by sparse single spikes from a few neurons; successively evolve during network development toward tonic network-wide firing manifesting bursting events at 81 DIV and that stabilize by displaying large bursting events propagating in the network at 90 DIV. These recordings were obtained without the support of astrocyte co-cultures as it was previously reported (Odawara et al., 2014) and confirm a specific effect on network development induced by the PDLO coating.

By looking at the firing rate at 90 DIV, it is striking to observe a much higher activity for neurons grown on PDLO coated substrates compared to a suppression of the activity for the PEI coated ones. This suppression can be associated with the significant decrease of the previously discussed PSD-95 expression, which might be the result of differences in synapse stabilization and formation rates, variation in synapse lose rates, or both (Okabe et al., 1999). It should also be noted that the high spiking activity observed in PDLO coated samples, combined with a prominent synchronous activity, i.e., synchronous bursts, promotes by itself the stabilization and the growth of newly formed synapses during development (Ben-Ari, 2001).

Over 3 months of network development, we report that both types of networks grown on PEI and PDLO-coated substrates show skewed lognormal-like distributions of their firing frequency for different time-points over their development, i.e., at 8, 81, and 90 DIV. Interestingly, such lognormal-like distribution of the neuronal firing frequencies was recently reported for *in vivo* neuronal recordings in different brain circuits, on monkeys and rodents (Buzsáki and Mizuseki, 2014) and also in our previous study on rat cultures coupled to 4096-electrode arrays (Amin et al., 2015). Here we report for the first time that such distribution is also observed *in vitro* on human-derived neurons and support the hypothesis that the lognormal-like distribution might be a general physiological attractor for the spikes contributions of neurons within networks. Our results show that these skewed lognormal-like distributions of the neuronal firing frequency within the network are shifted or concomitant for PEI and PDLO grown networks, depending on the network maturation. In particular, the shift observed between 81 and 90 DIV in the distribution for networks grown on PDLO substrate might be interpreted as an indicator of sufferance of the network. However, this is unlikely the case as corroborated with healthy neuritic processes indicated by the optical read-outs and significant increase of active electrodes. Rather, as suggested by Teramae and Fukai (2014), changes in distributions observed during development might be linked to changes in the distribution of the synaptic weights and might underlie activity-dependent synaptic changes during development. Furthermore, another possibility for the source of asymmetrical distribution of firing rate reported in cultures grown on PDLO vs. PEI, might be due to the imbalance in the excitatory and inhibitory synaptic drive during network activity (Yassin et al., 2010). The latter hypothesis is inline with our findings on the (V-GLUT/V-GAT) ratio that indicates such a balancing difference between networks grown on PDLO-/PEI-coated substrates.

It is also worth noting that our experiments were performed in neuronal networks that, albeit the long incubation period of 90 DIV, could be still relatively immature, as it has been previously reported based on the gene expression profile of iCell neurons observed in neonatal prefrontal cortex (Dage et al., 2014). In this respect, our results suggest that the developmental time needed to obtain functional and mature human-derived neuronal networks might be even longer than 3 months.

### Human-derived neuronal networks can respond to electrical stimulation

Responsiveness to electrical stimulation is an electrophysiological property of neuronal networks. Our results show that electrically elicited network-wide responses are observed only for iCell neuronal networks grown on PDLO coated substrates and only after 3 months of culture. Additionally, our analysis of the evoked waveforms revels that the repertoire of the electrically evoked signals does not show significant differences in the waveforms with respect to the ones observed for cultured hippocampal networks from rat embryos.

The long developmental time needed to the network to respond to electrical stimuli might denote a slow establishment of effective functional connections among neurons in these cultures. On the other hand, the lack of responsiveness to electrical stimuli over 3 months for cultures grown on PEI substrates is unclear and might be related to the prominence of inhibition of these networks as observed with the quantification of the V-GLUT/V-GAT ratio.

In addition, for responsive networks we also observed two classes of evoked spiking responses for the different on-chip stimulations sites, i.e., either lasting less than 100 ms, or, surprisingly, lasting more than 500 ms and with also a longer time-delay of the first spike. In our experience, such a long-lasting response to electrical stimuli was not previously observed on cultures of rodent's neurons, either from wild-type mice or rats. There are three possible interpretations for these long-lasting responses. First, they might result from a heterogeneous development of the effective connectivity within the network; hence, causing a delay in the neuronal information flow conveyed thoroughly evoked spiking activity. Another reason might be related to recurrent activations of synaptic excitation (Lau and Bi, 2005) and might underlie the dynamical properties of a subset of the network that provides to some extent a specific subpopulation of neurons in the same network. Finally, it cannot be excluded that this type of responses might indicate typical features of human neuronal networks that provide their own importance and function for the maintenance of the physiological conditions during development. However, the understanding of the mechanisms behind these network responses requires further studies to be performed.

## Conclusions and outlooks

Overall, the results of this work represent in our opinion a relevant step in the still young and growing field of research on networks formed by human-derived neurons. Indeed, the assessment of the functional properties emerging from these networks and the optimization of their cell culture conditions are two major requirements for the potential exploitation of this emerging biotechnology.

In particular, toward the development of cell-culture assays for drug screenings and toxicology, here we have shown the combined use of two emerging technologies, namely hiPSC-derived neuronal cultures and large-scale electrode array platforms, i.e., CMOS-MEA. Our morphological and electrophysiological results allowed us to pinpoint the key role of surface functionalization for supporting the development of human-derived neuronal networks grown *in vitro* up to several months. Surface functionalization of multielectrode arrays is a known 2-fold problem, with relevant implications to the culture viability and to the electrical recording performance (Wheeler et al., 1999; Jungblut et al., 2009; Sun et al., 2012). Our results show that PDLO coated substrates appear to be better adapted to the growth of human-derived neurons and this functionalization allows for the recording of both the spontaneous and evoked spiking activities on CMOS-MEAs expressed by these networks. However, the reason of the failure of other tested functionalization needs to be further investigated.

Our results also highlight that significant challenges on human-derived neurons remain to be overcome. Indeed, even after 3 months of culture, our results show that the emerging network properties are not completely comparable with what has been observed on neuronal networks prepared from mice or rats neuronal cultures. This might be interpreted as an unmet sufficient level of maturation of the network or as intrinsic functional properties of networks formed by these human-derived neurons. Based on the results presented in this study, we consider the first hypothesis as the most plausible. Indeed, it has been already reported that human iCell neurons are developing slower than murine neuronal cultures (Ohara et al., 2015). To a certain extent, the need of a long maturation might be due to the need of further optimizing the nutritive components and serum protein content in the supplemented media (Dodla et al., 2010). Additionally, it has to be reminded that these human neurons were prepared from pre-differentiated cryopreserved stocks, a condition that may induce the need of a longer recovery period to develop a physiological neuritogenesis.

In light of this, new *in vitro* differentiation strategies and protocols of hiPSCs have to be advanced, given their role to generate more stringent functionally differentiated neurons for their final appropriate use in research and therapy. Notably, neuronal responsiveness to pharmaceutical compounds has been reported to be maturation-state-dependent (Falcão et al., 2006); therefore, it is crucial to profoundly characterize the maturation profile of these human derived neurons in order to increase their responses fidelity and reproducibility for any potential pharmacological and toxicological tests. Therefore, perspective studies must be performed over long-time (several months), to investigate and optimize the maturation profile of these human derived neurons. For instance, studies aiming at quantify the expression of two GABA transporters, i.e., Na^+^-K^+^-2Cl^−^co-transporter (NKCC1) and the K^+^-Cl^−^co-transporter (KCC2), that play a crucial role in regulating the intracellular chloride of neurons during developmental stages (Ben-Ari, 2002) would provide important insights on the network development of these neurons.

## Ethics statement

iCell neurons used in this study were purchased from CDI (USA). These human cells differentiated from stable iPS cell lines that were derived from human peripheral blood or fibroblasts. Human blood or fibroblasts were collected under a donor informed consent contemplating ethical requirements for broad research and commercial use. Cellular reprogramming was performed at CDI using intellectual property and protocols owned by, or licensed to, CDI.

## Author contributions

HA, FM, and LB designed the research project. HA performed experiments. HA, AM, TN analyzed data. SZ developed Software and server-client tool. HA, and LB wrote the paper. All co-authors reviewed the paper.

### Conflict of interest statement

LB is a co-founder of 3Brain GmbH, but without any executive role in management, strategic planning or operation of the company. 3Brain GmbH and LBe act independently in their research and commercial activities.

## References

[B1] Amin H. Maccione A. Zordan S. Nieus T. Berdondini L. (2015). High-Density MEAs reveal lognormal firing patterns in neuronal networks for short and long term recordings, in 2015 7th International IEEE/EMBS Conference on Neural Engineering (NER) (Montpellier: IEEE), 1000–1003.

[B2] Ben-Ari Y. (2001). Developing networks play a similar melody. Trends Neurosci. 24, 353–360. 10.1016/S0166-2236(00)01813-0 11356508

[B3] Ben-Ari Y. (2002). Excitatory actions of Gaba during development: the nature of the nurture. Nat. Rev. Neurosci. 3, 728–739. 10.1016/S0166-2236(00)01813-0 12209121

[B4] Berdondini L. Imfeld K. Maccione A. Tedesco M. Neukom S. Koudelka-Hep M. (2009). Active pixel sensor array for high spatio-temporal resolution electrophysiological recordings from single cell to large scale neuronal networks. Lab. Chip. 9, 2644–2651. 10.1039/b907394a 19704979

[B5] Berdondini L. Overstolz T. de Rooij N. F. Koudelka-Hep M. Wany M. Seitz P. (2001). High-density microelectrode arrays for electrophysiological activity imaging of neuronal networks, in ICECS 2001. 8th IEEE International Conference on Electronics, Circuits and Systems (Cat. No.01EX483), Vol. 3 (Malta: IEEE), 1239–1242

[B6] Buzsáki G. Mizuseki K. (2014). The log-dynamic brain: how skewed distributions affect network operations. Nat. Rev. Neurosci. 15, 264–278. 10.1038/nrn3687 24569488 PMC4051294

[B7] Chatzidaki A. Fouillet A. Li J. Dage J. Millar N. S. Sher E.. (2015). Pharmacological characterisation of Nicotinic Acetylcholine receptors expressed in human iPSC-derived neurons. PLoS ONE 10:e0125116. 10.1371/journal.pone.0125116 25906356 PMC4408108

[B8] Chiappalone M. Bove M. Vato A. Tedesco M. Martinoia S. (2006). Dissociated cortical networks show spontaneously correlated activity patterns during *in vitro* development. Brain Res. 1093, 41–53. 10.1016/j.brainres.2006.03.049 16712817

[B9] Dage J. L. Colvin E. M. Fouillet A. Langron E. Roell W. C. Li J. Mathur S. X.. (2014). Characterisation of Ligand- and Voltage-Gated ion channels expressed in human iPSC-derived forebrain neurons. Psychopharmacology 231, 1105–1124. 10.1007/s00213-013-3384-2 24429870

[B10] Dayan P. Abbott L. F. (2005). Theoretical Neuroscience: Computational and Mathematical Modeling of Neural Systems. The MIT Press Available onine at: http://dl.acm.org/citation.cfm?id=1205781

[B11] Dodla M. C. Jennifer M. Steven L. S. (2010). Role of Astrocytes, Soluble factors, cells adhesion molecules and neurotrophins in functional synapse formation: implications for human embryonic stem cell derived neurons. Curr. Stem Cell Res. Ther. 5, 251–260. 10.2174/157488810791824520 20214556

[B12] Ehrlich I. Matthew K. Simon R. Roberto M. (2007). PSD-95 Is required for activity-driven synapse stabilization. Proc. Natl. Acad. Sci.U.S.A. 104, 4176–4181. 10.1073/pnas.0609307104 17360496 PMC1820728

[B13] El-Husseini A. E. Schnell E. Chetkovich D. M. Nicoll R. A. Bredt D. S. (2000). PSD-95 involvement in maturation of excitatory synapses. Science 290, 1364–1368. 10.1126/science.290.5495.1364 11082065

[B14] Espuny-Camacho I. Kimmo A. M. David G. Daniele L. Anja H. Bonnefont J.. (2013). Pyramidal neurons derived from human pluripotent stem cells integrate efficiently into mouse brain circuits *in vivo*. Neuron 77, 440–456. 10.1016/j.neuron.2012.12.011 23395372

[B15] Eversmann B. Jenkner M. Hofmann F. Paulus C. Brederlow R. Holzapfl B. Fromherz P. (2003). A 128 X 128 Cmos biosensor array for extracellular recording of neural activity. IEEE J. Solid State Circuits 38, 2306–2317. 10.1109/JSSC.2003.819174

[B16] Falcão A. S. Fernandes A. Brito M. A. Silva R. F. Brites D. (2006). Bilirubin-induced immunostimulant effects and toxicity vary with neural cell type and maturation state. Acta Neuropathol. 112, 95–105. 10.1007/s00401-006-0078-4 16733655

[B17] Franks W. Heer F. McKay I. Taschini S. Sunier R. Hagleitner C. (2003). CMOS Monolithic microelectrode array for stimulation and recording of natural neural networks, in TRANSDUCERS'03. 12th International Conference on Solid-State Sensors, Actuators and Microsystems. Digest of Technical Papers (Cat. No.03TH8664), Vol. 2 (Boston, MA: IEEE), 963–966.

[B18] Gerstein G. L. Kiang N. Y. (1960). An approach to the quantitative analysis of electrophysiological data from single neurons. Biophys. J. 1, 15–28. 13704760 10.1016/s0006-3495(60)86872-5 PMC1366309

[B19] Gill J. K. Chatzidaki A. Ursu D. Sher E. Millar N. S. (2013). Contrasting properties of α7-selective orthosteric and allosteric agonists examined on native nicotinic acetylcholine receptors. PLoS ONE 8:e55047. 10.1371/journal.pone.0055047 23383051 PMC3558472

[B20] Haythornthwaite A. Stoelzle S. A. Hasler A. Kiss A. Mosbacher J. George M.. (2012). Characterizing human ion channels in induced pluripotent stem cell-derived neurons. J. Biomol. Screen. 17, 1264–1272. 10.1177/1087057112457821 22923790

[B21] Hunt C. A. Schenker L. J. Kennedy M. B. (1996). PSD-95 Is associated with the postsynaptic density and not with the presynaptic membrane at forebrain synapses. *J*. Neurosci. 16, 1380–1388. 8778289 10.1523/JNEUROSCI.16-04-01380.1996 PMC6578559

[B22] Ide A. N. Andruska A. Boehler M. Wheeler B. C. Brewer G. J. (2010). Chronic network stimulation enhances evoked action potentials. J. Neural Eng. 7:16008. 10.1088/1741-2560/7/1/016008 20083862 PMC3775841

[B23] Jimbo Y. Kawana A. (1992). Electrical stimulation and recording from cultured neurons using a planar electrode array. Bioelectrochem. Bioenerg. 29, 193–204. 10.1016/0302-4598(92)80067-Q

[B24] Jungblut M. Knoll W. Thielemann C. Pottek M. (2009). Triangular Neuronal networks on microelectrode arrays: an approach to improve the properties of low-density networks for extracellular recording. Biomed. Microdevices 11, 1269–1278. 10.1007/s10544-009-9346-0 19757074 PMC2776171

[B25] Lau P. M. Bi G. Q. (2005). Synaptic mechanisms of persistent reverberatory activity in neuronal networks. Proc. Natl. Acad. Sci. U.S.A. 102, 10333–10338. 10.1073/pnas.0500717102 16006530 PMC1177363

[B26] Levinson J. N. El-Husseini A. (2005). New players tip the scales in the balance between excitatory and inhibitory synapses. Mol. Pain 1:12. 10.1186/1744-8069-1-12 15813960 PMC1079938

[B27] Llinas R. (1988). The intrinsic electrophysiological properties of mammalian neurons: insights into central nervous system function. Science 242, 1654–1664. 10.1126/science.3059497 3059497

[B28] Maccione A. Gandolfo M. Massobrio P. Novellino A. Martinoia S. Chiappalone M. (2009). A novel algorithm for precise identification of spikes in extracellularly recorded neuronal signals. J. Neurosci. Methods 177, 241–249. 10.1016/j.jneumeth.2008.09.026 18957306

[B29] Maccione A. Gandolfo M. Tedesco M. Nieus T. Imfeld K. Martinoia S.. (2010). Experimental investigation on spontaneously active hippocampal cultures recorded by means of high-density meas: analysis of the spatial resolution effects. Front. Neuroeng. 3:4. 10.3389/fneng.2010.00004 20485465 PMC2871691

[B30] Matsuzawa M. Weight F. F. Potember R. S. Liesi P. (1996). Directional neurite outgrowth and axonal differentiation of embryonic hippocampal neurons are promoted by a neurite outgrowth domain of the B2-chain of laminin. Int. J. Dev. Neurosci. 14, 283–295. 10.1016/0736-5748(96)00014-7 8842805

[B31] Nam Y. Wheeler B. C. (2011). *In vitro* microelectrode array technology and neural recordings. Crit. Rev. Biomed. Eng. 39, 45–61. 10.1615/CritRevBiomedEng.v39.i1.40 21488814

[B32] Odawara A. Saitoh Y. Alhebshi A. H. Gotoh M. Suzuki I. (2014). Long-term electrophysiological activity and pharmacological response of a human induced pluripotent stem cell-derived neuron and astrocyte co-culture. Biochem. Biophys. Res. Commun. 443, 1176–1181. 10.1016/j.bbrc.2013.12.142 24406164

[B33] Ohara Y. Koganezawa N. Yamazaki H. Roppongi R. T. Sato K. Sekino Y. Shirao T. (2015). Early-stage development of human induced pluripotent stem cell-derived neurons. J. Neurosci. Res. 93, 1804–1813. 10.1002/jnr.23666 26346430 PMC5049656

[B34] Okabe S. Kim H. D. Miwa A. Kuriu T. Okado H. (1999). Continual remodeling of postsynaptic density and its regulation by synaptic activity. Nat. Neurosci. 2, 804–811. 10.1038/12175 10461219

[B35] Orloff J. Douglas F. Pinheiro J. Levinson S. Branson M. Chaturvedi P. Ette E.. (2009). The future of drug development: advancing clinical trial design. Nat. Rev. 8, 949–957. 10.1038/nrd3025 19816458

[B36] Prange O. Wong T. P. Gerrow K. Wang Y. T. El-Husseini A. (2004). A balance between excitatory and inhibitory synapses is controlled by PSD-95 and neuroligin. Proc. Nat. Acad. Sci. U.S.A. 101, 13915–1320. 10.1073/pnas.0405939101 15358863 PMC518853

[B37] Prodanov D. Heeroma J. Marani E. (2006). Automatic morphometry of synaptic boutons of cultured cells using granulometric analysis of digital images. J. Neurosci. Methods 151, 168–177. 10.1016/j.jneumeth.2005.07.011 16157388

[B38] Rajamohan D. Matsa E. Kalra S. Crutchley J. Patel A. George V.. (2013). Current status of drug screening and disease modelling in human pluripotent stem cells. BioEssays 35, 281–298. 10.1002/bies.201200053 22886688 PMC3597971

[B39] Sans N. Petralia R. S. Wang Y. X. Blahos J. Hell J. W. Wenthold R. J. (2000). A developmental change in NMDA receptor-associated proteins at hippocampal synapses. J. Neurosci. 20, 1260–1271. 10648730 10.1523/JNEUROSCI.20-03-01260.2000 PMC6774158

[B40] Schneider C. A. Rasband W. S. Eliceiri K. W. (2012). NIH Image to ImageJ: 25 years of image analysis. Nat. Methods 9, 671–675. 10.1038/nmeth.2089 22930834 PMC5554542

[B41] Shoham S. Fellows M. R. Normann R. A. (2003). Robust, automatic spike sorting using mixtures of Multivariate T-Distributions. J. Neurosci. Methods 127, 111–122. 10.1016/S0165-0270(03)00120-1 12906941

[B42] Sun Q. Turrigiano G. G. (2011). PSD-95 and PSD-93 play critical but distinct roles in synaptic scaling up and down. J. Neurosci. 31, 6800–6808. 10.1523/JNEUROSCI.5616-10.2011 21543610 PMC3113607

[B43] Sun Y. Huang Z. Liu W. Yang K. Sun K. Xing S.. (2012). Surface coating as a key parameter in engineering neuronal network structures *in vitro*. Biointerphases 7, 1–14. 10.1007/s13758-012-0029-7 22589072

[B44] Taft C. E. Turrigiano G. G. (2014). PSD-95 promotes the stabilization of young synaptic contacts. Philos. Trans. R. Soci. Lond. B Biol. Sci. 369:20130134. 10.1098/rstb.2013.0134 24298137 PMC3843867

[B45] Takahashi K. Tanabe K. Ohnuki M. Narita M. Ichisaka T. Tomoda K.. (2007). Induction of pluripotent stem cells from adult human fibroblasts by defined factors. Cell 131, 861–872. 10.1016/j.cell.2007.11.019 18035408

[B46] Teramae B. J. Fukai T. (2014). Computational implications of lognormally distributed synaptic weights, in Proceedings of the IEEE, Vol. 102, 500–512. 10.1109/jproc.2014.2306254

[B47] van den Bogaart G. Meyenberg K. Risselada H. J. Amin H. Willig K. I. Hubrich B. E. Dier M.. (2011). Membrane protein sequestering by ionic protein–lipid interactions. Nature 479, 552–555. 10.1038/nature10545 22020284 PMC3409895

[B48] Wagenaar D. A. Pine J. Potter S. M. (2004). Effective parameters for stimulation of dissociated cultures using multi-electrode Arrays. J. Neurosci. Methods 138, 27–37. 10.1016/j.jneumeth.2004.03.005 15325108

[B49] Wagenaar D. A. Pine J. Potter S. M. (2006). An extremely rich repertoire of bursting patterns during the development of cortical cultures. BMC Neurosci. 7:11. 10.1186/1471-2202-7-11 16464257 PMC1420316

[B50] Wheeler B. C. Corey J. M. Brewer G. J. Branch D. W. (1999). Microcontact printing for precise control of nerve cell growth in culture. J. Biomech. Eng. 121, 73. 10080092 10.1115/1.2798045

[B51] Whitemarsh R. C. M. Strathman M. J. Chase L. G. Stankewicz C. Tepp W. H. Johnson E. A.. (2012). Novel application of human neurons derived from induced pluripotent stem cells for highly sensitive botulinum neurotoxin detection. Toxicol. Sci. 126, 426–435. 10.1093/toxsci/kfr354 22223483 PMC3307606

[B52] Wonders C. P. Anderson S. A. (2006). The origin and specification of cortical interneurons. Nat. Rev. Neurosci. 7, 687–696. 10.1038/nrn1954 16883309

[B53] Yassin L. Benedetti B. L. Jouhanneau J. S. Wen J. A. Poulet J. F. Barth A. L. (2010). An embedded subnetwork of highly active neurons in the neocortex. Neuron 68, 1043–1050. 10.1016/j.neuron.2010.11.029 21172607 PMC3022325

[B54] Ziff E. B. (1997). Enlightening the postsynaptic density. Neuron 19, 1163–1174. 10.1016/S0896-6273(00)80409-2 9427241

