# Supplementary material for: Electrical Responses and Spontaneous Activity of Human iPS-Derived Neuronal Networks Characterized for 3-month Culture with 4096-Electrode Arrays
Source: Front Neurosci. 2016 Mar 30;10:121. doi: 10.3389/fnins.2016.00121 (PMC4811967; doi:10.3389/fnins.2016.00121)
Supplement: Supplementary file 2 [file DataSheet1.DOCX]

Supplementary Material

Electrical responses and spontaneous activity of human iPS-derived neuronal networks characterized for three-month culture with 4096-electrode arrays

**Hayder Amin*, Alessandro Maccione, Federica Marinaro, Stefano Zordan, Thierry Nieus, and Luca Berdondini**

*** Correspondence:** Hayder Amin: hayder.amin@iit.it

# Supplementary Figures and Videos

## Supplementary Figures

**Supplementary Figure 1:** Confocal micrographs of human-derived neurons grown on PLO-coated substrate show clustered and bundled-like morphology. Scale bar represents 30 µm.

**Supplementary Figure 2: The effect of biochemical adhesion molecules on the PSD-95 expression in low-density iCell neuronal cultures. (A)** Confocal micrographs of low-dense neurons (40’000 cells per CMOS-MEA “BioChip 4096E”), grown on PDLO and PEI substrates are fixed and stained at 90 DIV for MAP-2, NeuN, and PSD-95. Cross-sections along the indicated regions elucidate the intensity and density of PSD-95 constructs. To quantify the puncta of PSD-95 constructs, images were processed with the granulometric filtering (see Materials and Methods, and **Figure 2C**). Blue and red cross-sections depict the fluorescence and the corresponding filtered intensities, respectively, thus indicating a significant higher puncta construct of PSD-95 in neurons grown on PDLO, compared to the lower puncta in those grown on PEI. The position of PSD-95 puncta was defined above an arbitrary offset, i.e. 0.07, here indicated by grey bars. Scale bars represent 10 µm. **(B)** 3D surface plots of full areas of the indicated micrographs in (A) displaying the pixel intensities in pseudo color image. Red cross-sections illustrate in 3D, the position of the quantified intensity and density of PSD-95 as in 2D images in (A).

**Supplementary Figure 3: The effect of biochemical adhesion molecules on V-GLUT/V-GAT ratio in iCell neuronal cultures**. Bar graph showing the ratio V-GLUT/VGAT significantly reduced (1.53 ± 0.08)-fold for neurons grown on PEI-coated substrates, compared to (4.2 ± 0.18)-fold for neurons seeded on PDLO substrates (n=3, at least 9 image fields per sample, ***p < 0.001).

**Supplementary Figure 4: Activity analysis of high-resolution electrical read-outs with CMOS-MEAs**. Activity maps obtained with the 4096 CMOS-MEA show the developmental changes in the spontaneous electrical activity of human-derived neurons over maturation (14, 28, 61, 73, 81, and 90 DIV) with respect to the first recorded time-set at 8 DIV. A prominent increase in the firing activity (as indicated by red pixels in the variation maps) is observed at 81 and at 90 DIV for neuronal networks grown on PDLO coated substrates, whilst the ones on PEI display a modest increase of the firing activity at 73 DIV, followed by a substantial decay. Color-code bar indicates the percentage of variation of firing rates for all recorded phases in respect to 8 DIV, where red is for increasing activity, and blue for decreasing activity.

**Supplementary Figure 5: The mean (µ) analysis of the Gaussian fit distributions**. (A) The position of the Gaussian fit peak of each recording phase (8, 81, and 90 DIV) showing the frequency shift from high toward low rates. (B) Similarly, as in (A), the positions of the Gaussian fit peaks for three recorded phases during development are unchanged, indicating no frequency shift occurred. In (A) and (B), Arrows on the y-axis show the shift directions on the logarithmic frequency scale, where (↑) refers to the right, and (↓) refers to the left.

**Supplementary Figure 6: Sampling frequency analysis of electrically evoked spike waveforms**. Waveforms of extracellular spontaneous activity recorded with two sampling rates, namely 7 kHz and 22 kHz. No significant differences are noticed. The sorting of waveforms into units performed on different sampling frequencies, also show identical results, where exemplary multi-units are detected in ch 05-63 and only single-unit in ch 03-61.

## Supplementary Video

**Supplementary Video 1: Movie of an evoked electrical response in a human iCell neuronal network coupled to 4096-electrode array.** The electrical stimulus is delivered with an on-chip electrode connected to a Plex-Stim Electrical Stimulator 2.0 System (Plexon Inc., USA). This stimulus is part of a sequence of electrical stimuli that were applied at low-frequency (0.2 Hz), and consisting in biphasic current stimuli (300 µs per phase, positive phase first, peak-to-peak amplitude of 300 µA). More details on the protocol are provided in section 3.3 of the Materials and Methods.
