# Supplementary figures and images for: Electrical Responses and Spontaneous Activity of Human iPS-Derived Neuronal Networks Characterized for 3-month Culture with 4096-Electrode Arrays
Source: Front Neurosci. 2016 Mar 30;10:121. doi: 10.3389/fnins.2016.00121 (PMC4811967; doi:10.3389/fnins.2016.00121)

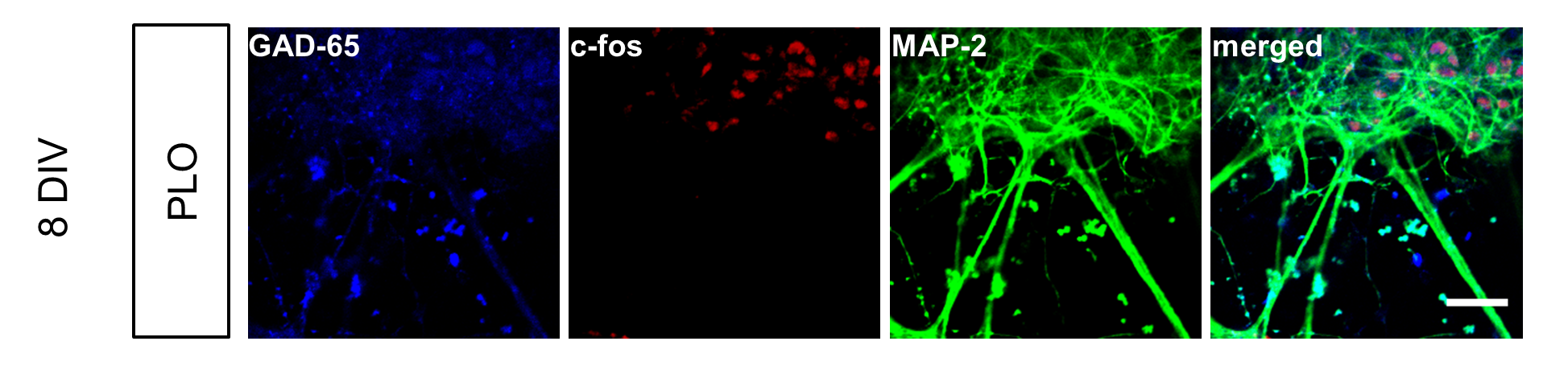

Supplement: Supplementary file 3 [file Image1.TIF]

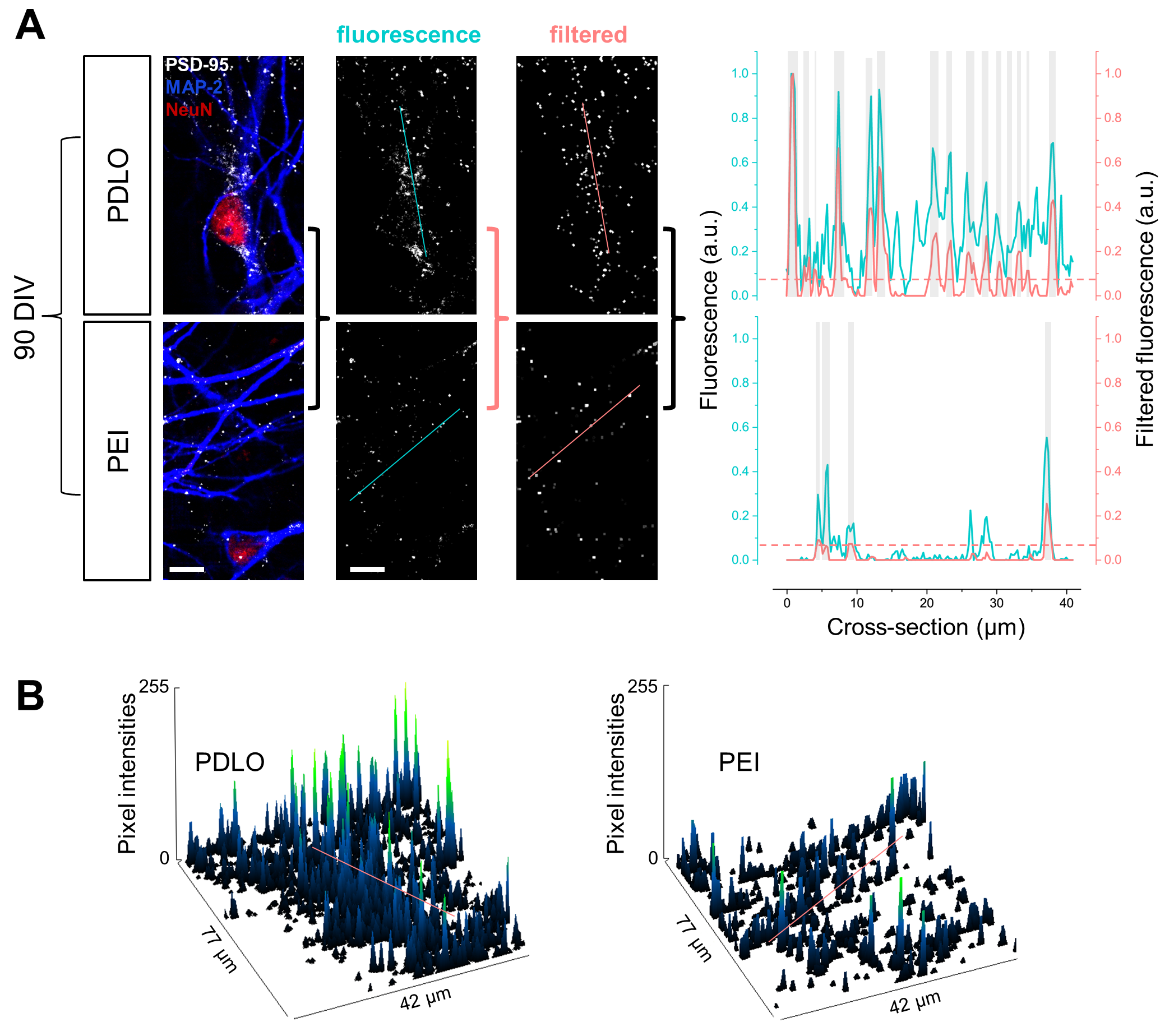

Supplement: Supplementary file 4 [file Image2.TIF]

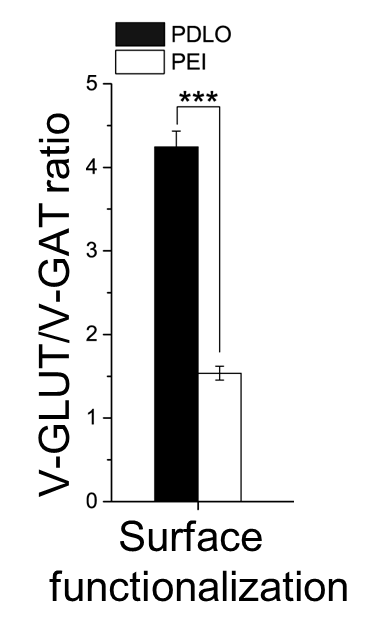

Supplement: Supplementary file 5 [file Image3.TIF]

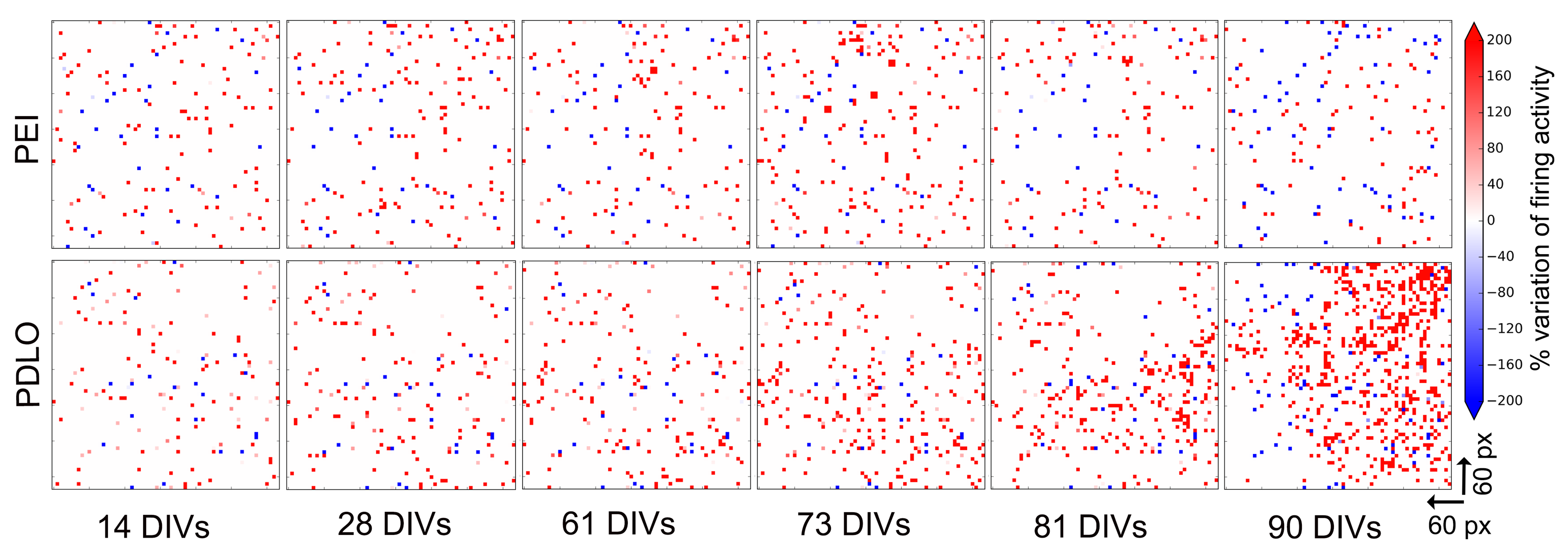

Supplement: Supplementary file 6 [file Image4.TIF]

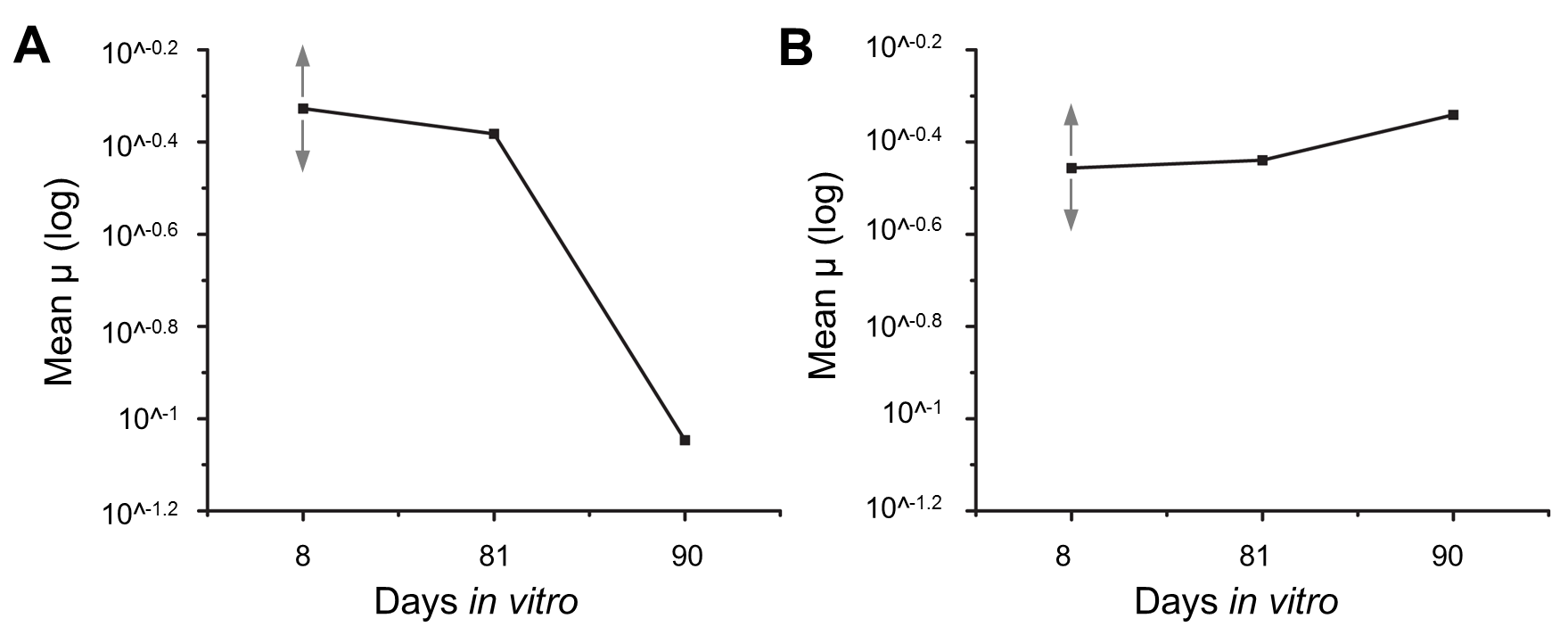

Supplement: Supplementary file 7 [file Image5.TIF]

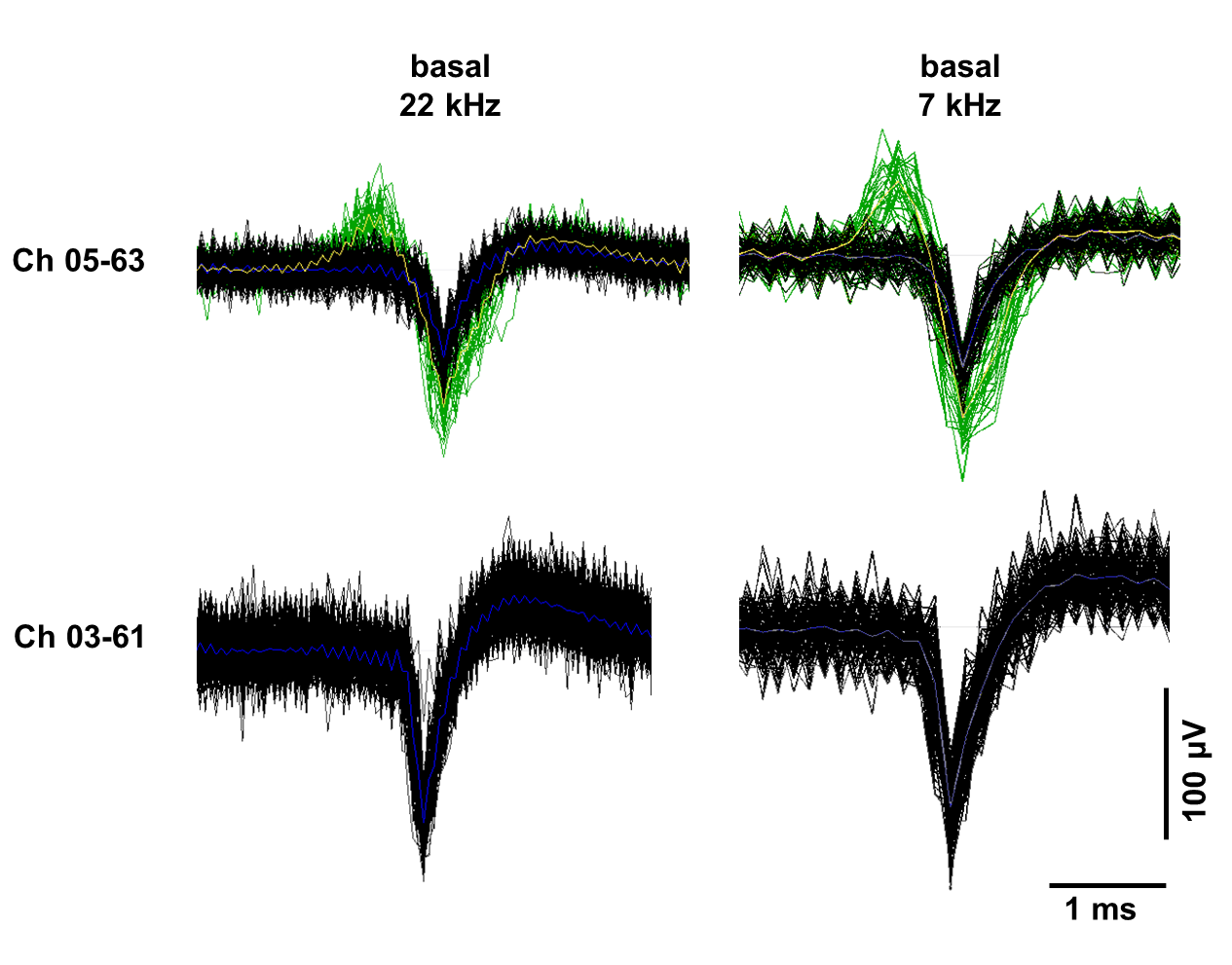

Supplement: Supplementary file 8 [file Image6.TIF]
